# The Geography of Fear: A Latitudinal Gradient in Anti-Predator Escape Distances of Birds across Europe

**DOI:** 10.1371/journal.pone.0064634

**Published:** 2013-05-28

**Authors:** Mario Díaz, Anders Pape Møller, Einar Flensted-Jensen, Tomáš Grim, Juan Diego Ibáñez-Álamo, Jukka Jokimäki, Gábor Markó, Piotr Tryjanowski

**Affiliations:** 1 Department of Biogeography and Global Change (BGC), Museo Nacional de Ciencias Naturales, CSIC, Madrid, Spain; 2 Lab. d'Ecologie, Systématique et Evolution, CNRS UMR 8079, University Paris-Sud, Orsay, France; 3 Cypresvej 1, DK-9700 Brønderslev, Denmark; 4 Palacký University, Department of Zoology and Lab. Ornithology, Olomouc, Czech Republic; 5 Department of Zoología, Fac. Ciencias, Campus Universitario de Fuentenueva s/n, University Granada, Granada, Spain; 6 University Lapland, Arctic Centre, Rovaniemi, Finland; 7 Rovaniemi University of Applied Sciences, Rantavitikka Campus, Rovaniemi, Finland; 8 Eötvös Loránd University, Behavioral Ecology Group, Department of Systematics, Zoology and Ecology, Budapest, Hungary; 9 Department of Plant Pathology, Corvinus University of Budapest, Budapest, Hungary; 10 Inst. Zoology, Poznań University of Life Sciences, Poznan, Poland; Liverpool John Moores University, United Kingdom

## Abstract

All animals flee from potential predators, and the distance at which this happens is optimized so the benefits from staying are balanced against the costs of flight. Because predator diversity and abundance decreases with increasing latitude, and differs between rural and urban areas, we should expect escape distance when a predator approached the individual to decrease with latitude and depend on urbanization. We measured the distance at which individual birds fled (flight initiation distance, FID, which represents a reliable and previously validated surrogate measure of response to predation risk) following a standardized protocol in nine pairs of rural and urban sites along a ca. 3000 km gradient from Southern Spain to Northern Finland during the breeding seasons 2009–2010. Raptor abundance was estimated by means of standard point counts at the same sites where FID information was recorded. Data on body mass and phylogenetic relationships among bird species sampled were extracted from the literature. An analysis of 12,495 flight distances of 714 populations of 159 species showed that mean FID decreased with increasing latitude after accounting for body size and phylogenetic effects. This decrease was paralleled by a similar cline in an index of the abundance of raptors. Urban populations had consistently shorter FIDs, supporting previous findings. The difference between rural and urban habitats decreased with increasing latitude, also paralleling raptor abundance trends. Overall, the latitudinal gradient in bird fear was explained by raptor abundance gradients, with additional small effects of latitude and intermediate effects of habitat. This study provides the first empirical documentation of a latitudinal trend in anti-predator behavior, which correlated positively with a similar trend in the abundance of predators.

## Introduction

The global distribution of biodiversity reflects higher degrees of specialization and interspecific interactions at low latitudes. There is a long tradition for linking these trends by viewing high tropical biodiversity as a consequence of interspecific interactions [1], [2]. For example, MacArthur [3] suggested explicitly that southern distribution limits of many species in the northern hemisphere likely depended on biotic interactions, while northern distributions more often were determined by abiotic factors. Studies of latitudinal trends in species diversity have shown consistent decreases with increasing latitude that is also repeated in predators, parasites and numerous other functional groups [e.g. 4–6]. Interestingly, not all groups show the same slope of species richness with latitude, implying that the abundance and species composition of different functional groups, and hence the intensity of interspecific interactions such as predation or competition, may change with latitude (reviewed by [7]).

Levels of defense are predicted to coevolve with levels of offense along productivity gradients such as the gradient from the arctic to the tropics [8]. Although predation constitutes an important interspecific interaction, relatively few studies have investigated latitudinal patterns in predation and anti-predator behavior [7]. Several studies have shown latitudinal changes in palatability [9] or chemical or mechanical defenses of prey [10], [11]. However, many studies relied on a sample of individuals from a single temperate and a single tropical site, thereby offering no replication (i.e., a case of pseudo-replication) and thus increasing the risk that between population differences were caused by factors unrelated to geographical position (e.g. [12]; reviewed in [7]). Furthermore, the generality of latitudinal gradients across taxa implies that processes underlying them should have evolved repeatedly through convergence. Recently, McKinnon et al. [13] showed a northwards decrease in nest predation rates of artificial nests along a latitudinal gradient of more than 3000 km in North America. Likewise, frogs *Rana temporaria* showed a strong latitudinal cline in anti-predator behavior in Sweden [14], and Møller and Liang [15] interpreted higher levels of fear (longer flight initiation distances -hereafter: FID- when approached by a researcher; see below) in Chinese as compared to European birds partly due to differences in predation risk. Although these studies provide important information, there is a need to extend them to include multiple model species along wide latitudinal gradients to test for the generality of patterns (i.e., meta-replication [16], [17]). Furthermore, it is also necessary to test the underlying assumption that differences in anti-predator behavior are related to differences in predation risk [18].

Animals in general show a range of fear responses that have important implications for understanding habitat use, but also life history, demography, interspecific interactions and conservation [18]–[21]. Flight initiation distance (hereafter FID) when approached by potential predators and other kinds of anti-predator behavior pose a problem of optimization of benefits associated with escape weighed against costs of disturbance (like stop feeding and/or energetic costs of flight), which may vary with ambient conditions. In general, FID constitutes a reliable measure of risk taking because it correlates with susceptibility to predation, even when the approaching potential predator was the researcher who measured such distances (see validation of flight distances as a meaningful proxy of risk taking in [22]–[24]). In addition, FID is highly repeatable for individuals among estimates [25], [26].

Another important aspect of risk taking is the effect of urbanization, especially given that several studies have found differences in predator densities between urban and non-urban habitats [27]–[31]. In fact, birds have considerably shorter FIDs in urban than in rural habitats [32], [33], mainly linked to differences in risk of predation and duration since urbanization [26]. These behavioral responses were largely independent of differences in habitat structure between urban and nearby rural sites [26], [34]. European species of birds with long FIDs have negative population trends as expected if there are costs associated with frequent disturbance by humans, dogs and other potential predators [34]. Thus, it is crucial to take this urbanization effect into account when investigating latitudinal gradients in FID and its relationships with predation risk.

**Figure 1:**
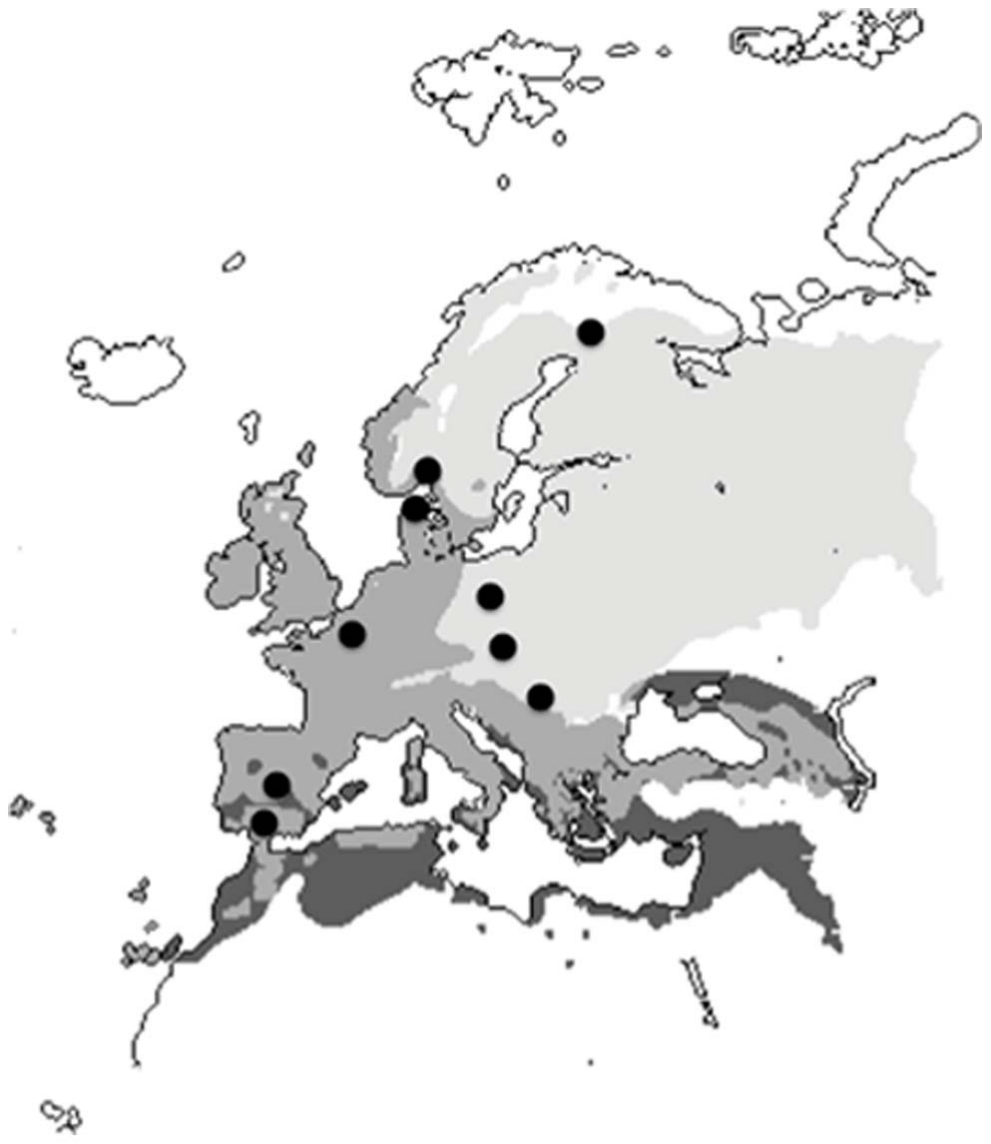
Location of the nine study sites for FID in Europe. Major climatic zones (subtropical, temperate and continental-subarctic, from darker to lighter shading) are also shown. For latitudes and city names see Table S1.

The objective of this study was to test for the existence and predictors of a latitudinal trend in FID of birds when being approached by a human, taken here as a methodologically proper surrogate for the risk of predation experienced by bird populations. We studied FIDs at nine different pairs of nearby rural and urban sites along a latitudinal gradient from Southern Spain to Northern Finland (Figure 1), allowing for repeated tests among independent populations within species. Bird census data from these sites [35] were used to test whether raptor abundance showed a similar latitudinal gradient. Because FID differs consistently between rural and urban populations [32], [33], we also tested if the latitudinal trend was consistent independently of habitat. Finally, we tested whether latitudinal gradients in FID were related to parallel gradients in raptor abundance. Overall, our main aim was to test whether different bird species showed consistent latitudinal patterns of anti-predator behavior in relation to spatial patterns of predation risk. If that was the case, this would be the first empirical documentation of such a latitudinal trend.

## Methods

### Ethic Statement

Recording of flight initiation distance does not require ethics approval or legal permits because it does not involve any important effect on animal welfare. The disturbance produced to birds by our methodology did not differ from standard “background” disturbance caused by humans: birds in our study sites, especially in the urban ones, are regularly flushed by citizens, dogs, cats, cars, etc., and by tourists, pets, agricultural workers and mushroom pickers in rural areas. Moreover, our presence in study sites involved no alteration of the habitat and it was very brief in time. We used public trails and roads, thus, there was no need to ask land managers for approval.

### Study Areas

Fieldwork was performed in nine cities, each paired with a nearby rural area, located along a large latitudinal gradient across Europe (Fig. 1). The distance between urban and rural study sites was 1–20 km. The benefits of this approach are two-fold: (1) neighboring study sites will share most potentially confounding environmental characteristics including weather, altitude, soil and many others; and (2) birds will not be prevented from moving between neighboring urban and rural habitats for distance reasons, although other factors such as philopatry and assortative mating may prevent such movement. All urban study sites included areas with multi-storey buildings, single family houses, roads and parks, while nearby rural areas had open farmland and woodland and did not contain continuous urban elements like multi-storey buildings, one family houses, roads and parks. This simple operational definition was also adopted in other studies [36]–[39], and our definitions of urban (percent of built-up area >50, building density >10/ha and residential human density >10/ha) and rural habitats (percent of built-up area 5–20, building density 2.5–10/ha, and residential human density 1–10/ha) follow the suggestion made by Marzluff et al. [39].

### Flight Initiation Distance

We recorded FIDs of birds when approached by a human during the breeding seasons 2009–2010 using a modified technique of that used by Blumstein [21]. A full description of the procedures and three different cross-validations of the data are reported elsewhere [24], [33], [34]. We tried to obtain data from as many species and individuals as possible by systematic searches of the study areas; at the same time, we tried to avoid sampling the same individual twice by moving to another site right after samples were taken. If the same general area was visited, only individuals of different species, sex or age than those sampled before were tested. In brief, when an individual bird had been located with a pair of binoculars, an observer moved at a normal walking speed towards the individual bird, while recording the number of 1 m steps [22]. The distance from the observer to the bird when it first took flight was recorded as the FID, while the ‘starting distance’ was the distance from where the observer started walking up towards the bird and the location of the bird. If the individual bird was positioned in the vegetation, the height above ground was recorded to the nearest meter (birds on the ground were assigned a height of zero). While recording these flight initiation distances, we also recorded date and time of day, and the sex and the age of the individual if external characteristics allowed sexing and aging with binoculars. Starting distances and FIDs were estimated as the Euclidian distances that equals the square root of the sum of the squared horizontal distances and the squared height above ground level [21].

Previous studies have shown that starting distance is strongly positively correlated with FID [21], thereby causing a problem of collinearity. We eliminated this problem of collinearity by searching habitats for birds with a pair of binoculars when choosing an individual for estimating flight initiation distance. In this way we assured that most individuals were approached from a distance of at least 30 m, thereby keeping starting distances constant across species.

We extracted information on mean body mass of adult birds of each species from Cramp & Perrins [40], as FIDs are consistently affected by bird size [27]. FID was weakly negatively related to starting distance in a Generalized Linear Model that included species, age, habitat, country and body mass as factors (partial *F = *34.99, df = 1, 12493, *P*<0.0001; FID and body mass log-transformed), explaining less than 0.3% of the variance (see [26] for a similar procedure). None of the results presented in this paper changed statistically when including starting distance as an additional variable, and we thus excluded this variable from all subsequent analyses for simplicity.

FIDs varied significantly among species, using one-way analysis of variance with log_10_-transformed flight distance as the response variable and species as a factor (*F = *29.58, df = 158, 12337, R^2^ = 0.33, *P*<0.0001, repeatability (R) following Becker [41] R = 0.27, SE = 0.03). Thus there was consistency in flight distance within species.

### Raptor Abundance

The breeding bird community at study sites was censused by means of standard point counts with unlimited recording distance [42]. Censuses were made during the springs 2009–2010 in both urban and rural habitats in all study locations at the same sites where FID information was recorded [35]. From these data we derived an index of abundance of raptors (mostly hawks *Accipiter* spp., kestrels *Falco* spp. and buzzards *Buteo buteo*) by summing the numbers of individuals detected at each site divided by the number of sampling points (data in [35]). Briefly, we placed 25–50 points in each urban and rural site at distances of at least 100 m between two consecutive points. The exact location of each point was determined with a GPS, allowing us to make the second census in exactly the same sites as the first census. The first census was made in early April in Southern Spain, delaying the census at higher latitudes so it was completed in Northern Finland in late May, and the second census was carried out three-four weeks later. The census started at local sunrise, while remaining five minutes at each point recording all birds seen or heard. Censuses started on separate days in urban and rural study plots ensuring that there was no difference in timing of censuses between habitats. The same observer made all surveys in each particular city and its paired rural site. Vegetation cover (trees, shrubs, herbs and grass) and cover with buildings and other man-made structures were evaluated in the field within 50 m of each survey point. We obtained very similar abundance indices when controlling or not controlling for differences in coverage for the three vegetation layers [35].

### Statistical Analyses

We log_10_-transformed FID, body mass and raptor abundance to achieve distributions that did not differ from normality. Differences in raptor abundance along the latitudinal gradient and between urban and rural sites were tested by means of General Linear Models (GLM). Relationships between FID and predictor (latitude, urban-rural habitat, and raptor abundance) and confounding (body size) variables were also tested by a GLM approach using the mean FID obtained for all individuals of the same species sampled in each study site. However, taxonomic units such as species cannot be considered statistically independent observations due to effects of common ancestry [43]. As species occupy a variable number of study sites [35], latitudinal or habitat effects could be partly due to phylogenetic effects mediated by species composition. To control for the phylogenetic relationship among the sampled species we used phylogenetic generalized least square regression (PGLS) models as implemented in R statistical environment (see [44], [45] for a similar approach). We used the R libraries *ape*, *MASS* and *mvtnorm* and the function *pglm3.1.r*. First, we estimated the phylogenetic scaling parameter lambda (λ), that varies between 0 (phylogenetic independence) and 1 (species’ traits covary in direct proportion to their shared evolutionary history; [43]). Then, we calculated the phylogenetically corrected correlation between the variables of interest after adjusting for phylogenetic effects through the estimated λ. We have used the most recent and comprehensive bird phylogeny available [46], after editing it to include different populations of the same species as polytomies with a constant small genetic distance of 1·10^−10^ between conspecific populations. This phylogeny considers *Corvus corone* and *C. cornix* as conspecifics, so that we have also assigned a genetic distance between them of 1·10^−10^. The R script and the edited phylogeny file are supplied as Files S1 and S2, respectively. Sequential (type I) models were fitted, including first the effect of body size to test for effects of latitude and habitat, and the sequential effects of body size and raptor abundance to ascertain whether latitudinal and habitat effects changed when raptor abundance effects were included first.

We evaluated the magnitude of associations between FID and predictor variables based on effect sizes and its 95% CI, as computed from *P* values of *t*-tests according to Cohen [47]. Cohen’s criteria were small (Pearson *r* <0.10, explaining <1% of the variance), intermediate (*r* = 0.11–0.49, 9–24% of the variance) and large (*r* >0.50, 25% or more of the variance) effect sizes.

## Results

### Summary Statistics for FID and Latitude

We obtained information on FID from 714 populations belonging to 159 species, with the total number of individual estimates of flight distance being 12,495 (Table S1). The number of estimates per species ranged from 1 to 1474, mean (SE) = 79 (9). There were 430 estimates from rural and 284 from urban populations. Latitude ranged from 37°N to 66°N, or 29°×111 km per degree = 3,220 km, with climate ranging from subtropical to subarctic. Mean FID in the 714 populations ranged from 1 to 180 m, mean (SE) = 11.46 m (1.03; back-transformed from log_10_-transformed data), with a highly significant effect of species (*F = *3.06, df = 158, 555, R^2^ = 0.47, *P*<0.0001).

### Raptor Density and Latitude

**Figure 2:**
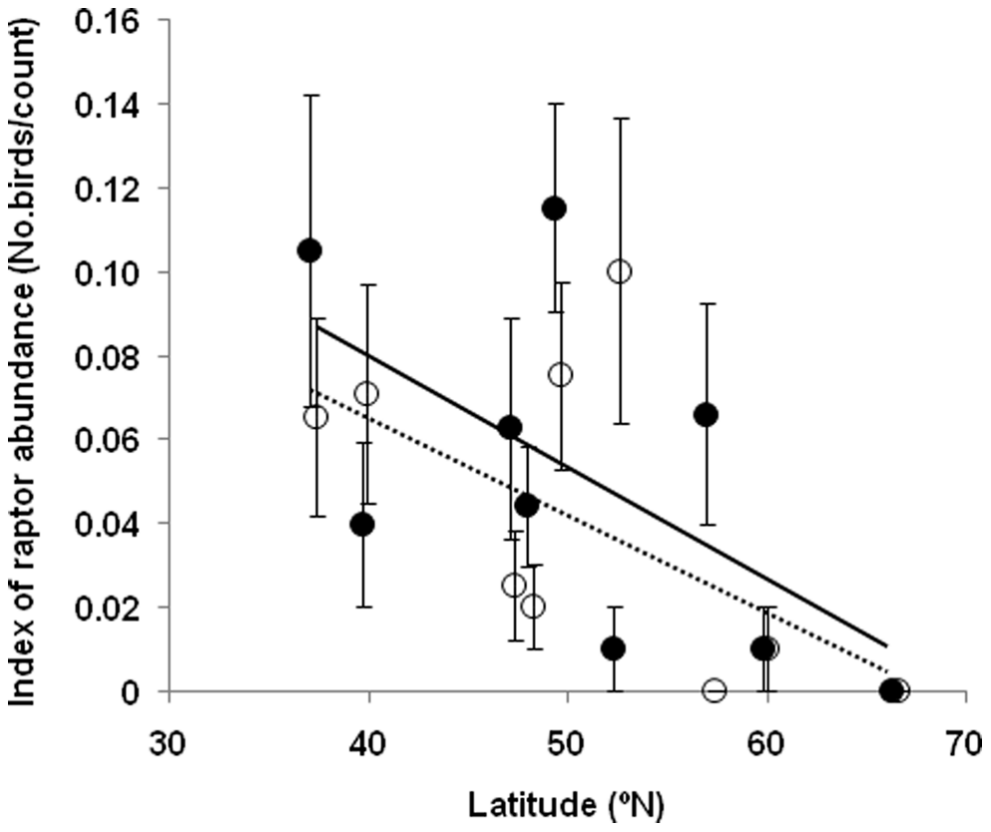
Mean (± SE) index of abundance of raptors in relation to latitude. Data are for paired rural (closed circles, continuous line) and urban (open circles, pointed line) sites. The index of abundance was estimated as the mean number of individuals detected at 25–50 point counts (depending on urban area size) each lasting five minutes. Lines are linear regression lines based on mean values. Symbols have been slightly displaced above (urban) and below (rural) real latitudes to improve clarity.

The abundance of raptors decreased significantly with increasing latitude when analyzing abundance at point counts (GLM; *F = *12.08, df = 1, 2425, *P = *0.0005) and was smaller in urban than in rural habitats (*F = *5.51, df = 1, 2425, *P = *0.019; least square mean for rural habitats: 0.0659 birds/census point (SE = 0.0080); least square mean for urban habitats: 0.0397 birds/census point (0.0077)). Mean density of raptors at the nine sites showed a significant trend with decreasing abundance at higher latitudes (Fig. 2; GLM: *F = *5.67, df = 1, 7, *P = *0.032) with a large effect size (*r* = 0.506; 95% CI: 0.052–0.787). This decreasing trend did not differ among paired urban and rural sites (*F = *1.19, df = 1, 7, *P = *0.293; Fig. 2).

### FID and Latitude

**Figure 3:**
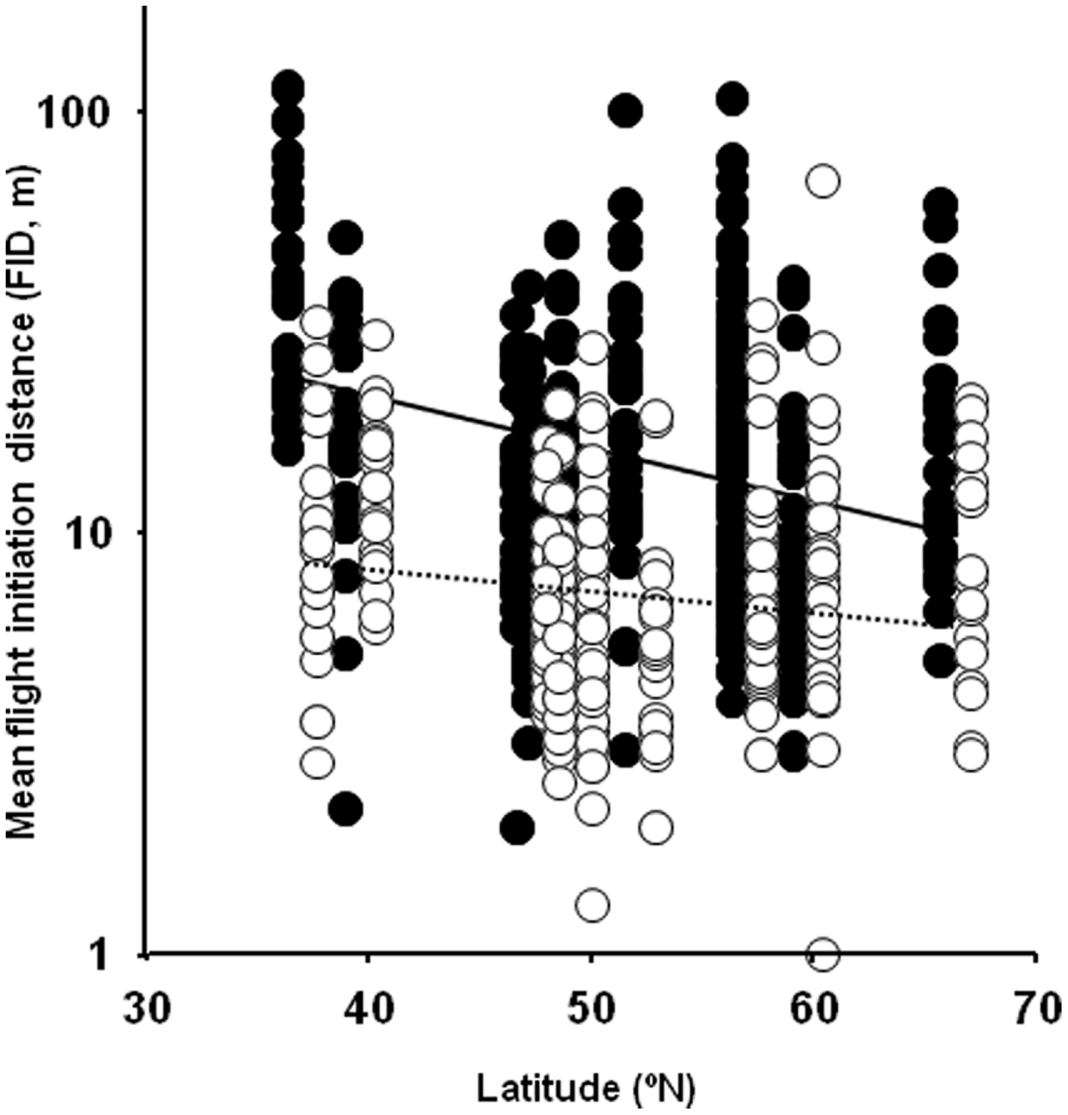
Mean FID (m) in relation to latitude for different populations and species of birds. Data are for paired rural (closed circles, continuous line) and urban (open circles, pointed line) sites. Lines are linear regression lines. Symbols have been slightly displaced above (urban) and below (rural) real latitudes to improve clarity.

Log-transformed mean FID of bird populations decreased with increasing latitude (Fig. 3), with an intermediate effect size (ca. 5%) after accounting for significant positive effect of body mass on FID as well as for phylogenetic effects (Table 1). Thus, anti-predator behavior decreased in intensity at higher latitudes. There was a highly significant difference in mean FID between populations of the same species from rural and urban habitats (mean (SE) rural = 13.34 m (1.05); urban = 7.12 m (1.05); paired t-test, *t* = −13.99, df = 229, *P*<0.0001), with a large effect size of 27% after accounting for body mass and phylogeny (Table 1). Finally, the difference in mean FID between rural and urban habitats decreased with increasing latitude (Fig. 3), with a small effect size (0.8%) after adjusting for the effect of body mass and phylogeny (Table 1).

**Table 1:** Flight initiation distance in relation to latitude, habitat (coded as 0 = rural or 1 = urban) and the interaction between latitude and habitat in European birds, after correcting for phylogenetic relationships among the sampled species by means of phylogenetic generalized least square regression (PGLS) models and for body size effects by mean of sequential (Type I) Sum of Squares (SS).

Source	df	SS	*F*	*P*	Effect size (*r*; 95%CI)
Body mass (log)	1	46.90	403.32	<0.0001	0.603 (0.554–0.648)
Latitude	1	7.44	64.01	<0.0001	0.219 (0.222–0.357)
Habitat	1	31.04	267.02	<0.0001	0.524 (0.470–0.577)
Latitude×Habitat	1	0.55	4.72	0.0174	0.090 (0.020–0.162)
Error	710	82.54			

The model had the statistics *F = *88.10, Adjusted R^2^ = 0.33, *P*<0.0001, AIC = 113.03. Phylogenetic scaling parameter (λ) = 0.472, *P*<0.0001. Effect sizes and their 95% CI were computed from *P* values of tests following [50].

Log-transformed mean FID of bird populations increased with increasing raptor abundance (F_1,712_ = 76.79, *P*<0.0001), with an intermediate effect size (ca 10%; *r* = 0.315; 95% CI: 0.247–0.380), after accounting for phylogenetic and body size effects by means of a sequential PGLS model including body size and raptor abundance. This model had the statistics *F*_2,711_ = 49.54, Adjusted R^2^ = 0.12, *P*<0.0001, AIC = 301.99, and phylogenetic scaling parameter (λ) = 0.472, *P*<0.0001. Latitudinal and habitat effects were still significant when accounting for the effects of raptor abundance (Table 2), but effect sizes decreased to small and intermediate, respectively (1.4 and 24%, respectively). The model including raptor abundance, latitude and habitat fitted significantly better to the data than the model not including this factor (ΔAIC = 113.03–106.84 = 6.19; Tables 1 and 2) and the model including only raptor abundance effects (ΔAIC = 301.99–106.84 = 195.15).

**Table 2:** Flight initiation distance in relation to latitude, habitat (coded as 0 = rural or 1 = urban) and the interaction between latitude and habitat in European birds, after correcting for phylogenetic relationships among the sampled species by means of phylogenetic generalized least square regression (PGLS) models, and for body size and raptor abundance effects by means of sequential (Type I) Sum of Squares (SS).

Source	df	SS	*F*	*P*	Effect size (*r*; 95%CI)
Body mass (log)	1	46.90	403.32	<0.0001	0.603 (0.554–0.648)
Raptor abundance (log)	1	12.99	113.59	<0.0001	0.374 (0.309–0.435)
Latitude	1	1.01	8.85	0.0016	0.118 (0.045–0.189)
Habitat	1	25.33	221.42	<0.0001	0.489 (0.431–0.543)
Latitude×Habitat	1	0.60	5.29	0.0124	0.094 (0.020–0.166)
Error	710	81.10			

The model had the statistics *F = *73.20, Adjusted *R* ^2^ = 0.34, *P*<0.0001, AIC = 106.84. Phylogenetic scaling parameter (λ) = 0.467, *P*<0.0001. Effect sizes and their 95% CI were computed from *P* values of tests following [50].

## Discussion

We have shown that flight initiation distance (FID) of birds when approached by a human, which constitutes an important component of anti-predator behavior, showed a clear-cut latitudinal gradient in Europe, after accounting statistically for factors influencing FID such as body size, habitat (urban or rural) or phylogenetic effects. This behavioral gradient was consistent among bird species and matched partially local changes in avian predator abundance. Overall, our study thus provides the first empirical documentation of a latitudinal trend in predator-prey interactions. Such a gradient may potentially influence the composition and structure of prey communities [7].

The latitudinal trend in anti-predator behavior paralleled latitudinal changes in raptor abundance, and varied depending on whether the study population was urban or rural, with smaller differences between rural and urban habitats at high latitudes, where overall raptor abundance was smaller. These findings show that anti-predator behavior consistently varied with latitude, apparently in response to latitudinal differences in predation risk [12]. Additionally, they highlight that the observed changes in anti-predator behavior detected in other studies due to the process of urbanization [30], [31] could depend on latitude too. However, predation risk, as measured by local estimates of raptor abundance, did not fully explain latitudinal and habitat differences in anti-predator behavior, as the most parsimonious model also included additional small effects of latitude *per se* and intermediate effects of habitat. In fact, the relationship between predator abundance and FID seemed to hold mainly for rural habitats along the sampled latitudinal gradient, whereas latitudinal changes in both FID and raptor abundance were much smaller in nearby urban sites (Figs. 2 and 3). We suggest that effects of other predators may account for additional variance.

Urban populations had consistently shorter flight distances than nearby rural populations, as found in previous studies of birds [32], [33], with the mean estimate being 7.1 m for urban birds and 13.3 m for rural birds, or an almost two-fold difference. There was an independent positive relationship between flight distance and body mass, with a large effect size after accounting for phylogenetic effects (Table 1). While many traits are correlated with body mass, thus making interpretation of this effect difficult, a greater difference in flight distance in large species may be attributed to smaller local population sizes, larger founder effects and elevated probability of local adaptation to urban conditions. Alternatively, large species have longer flight distances than small species [33], [48], and such large flight distances may be particularly energetically costly due to the disproportionately high costs of short flights that may reach a level 23 times higher than basal metabolic rate [49]. Thus, selection for adaptation to urban conditions will be particularly intense in large species due to frequent disturbance and flight, and relatively large reductions in flight distance of urban compared to rural populations in large species are consistent with this interpretation. Incidentally, this body size effect contributes to explain why urban areas are safer for prey, as avian predators are more affected by human disturbance in cities than their smaller-sized prey [48].

We predicted that interspecific interactions as reflected by the abundance of raptors are more important at southern latitudes, as the relative importance of abiotic (i.e. climatic) vs. biotic factors (i.e. competition, predation) on prey would decrease at lower latitudes. The observation that differences in FID between rural and urban habitats decreased towards the north (in the northern hemisphere) supports this prediction. Thus, our findings suggest that differences in abundance of raptors contributed to explain the difference in FID between rural and urban habitats along the latitudinal gradient across Europe. These findings are also consistent with the original suggestions by Janzen, Connell and MacArthur [1]–[3] that biotic interactions are important at lower latitudes, while abiotic factors are more important at higher latitudes.

In conclusion, we have shown that flight distance in birds, when approached by a human (an important component of anti-predator behavior) decreased with latitude, although varying among species and between rural and urban sites locally. Differences in flight distance between rural and urban habitats decreased with increasing latitude Overall, we have demonstrated for the first time a consistent spatial pattern of anti-predator behavior over a wide latitudinal and land-use gradient, with potential relevant effects on prey communities with different susceptibilities to predation.

## Supporting Information

Table S1 Summary statistics. Information on bird species and populations, study cities and countries, mean FID (m), SD FID (m), sample size (N), habitat: urban (1) or rural (0), latitude of study site (° N) and body mass (g) for the 714 sampled populations. See Material and methods for sources. Nomenclature follows [46] and population codes match those in file S2, where such populations are included as polytomies with a distance of 1·10^−10^.
(DOC)

File S1 R script for the phylogenetic generalized least square regression (PGLS) models. They were fitted to analyze relationships among FID, body mass, raptor abundance, latitude and habitat.
(DOCX)

File S2 Phylogeny of all European bird species edited to include the sampled populations as polytomies. Polytomies were indicated with a constant small genetic distance of 1·10^−10^ between conspecific populations.
(TXT)
