# Supplementary material for: The Geography of Fear: A Latitudinal Gradient in Anti-Predator Escape Distances of Birds across Europe
Source: PLoS One. 2013 May 28;8(5):e64634. doi: 10.1371/journal.pone.0064634 (PMC3665823; doi:10.1371/journal.pone.0064634)
Supplement: Table S1 — Summary statistics. Information on bird species and populations, study cities and countries, mean FID (m), SD FID (m), sample size (N), habitat: urban (1) or rural (0), latitude of study site (° N) and body mass (g) for the 714 sampled populations. See Material and methods for sources. Nomenclature follows [46] and population codes match those in file S2, where such populations are included as polytomies with a distance of 1·10−10. (DOC) [file pone.0064634.s001.doc]

**Table S1. Summary statistics.** Information on bird species and populations, study cities and countries, mean FID (m), SD FID (m), sample size (N), habitat: urban (1) or rural (0), latitude of study site (º N) and body mass (g) of the 714 sampled populations. See Material and methods for sources. Nomenclature follows [47] and population codes match the phylogeny file in which such populations are included as polytomies with a distance of 1x10^-10^.

| Species and population | city (country) | FID | SD FID | N | Habitat | Latitude | Body mass |
| --- | --- | --- | --- | --- | --- | --- | --- |
| Accipiter_nisus1 | Brønderslev (Denmark) | 36.14 |  | 1 | 0 | 57.2 | 204 |
| Accipiter_nisus2 | Paris (France) | 10 | 4.24 | 2 | 1 | 48.1 | 204 |
| Acrocephalus_palustris1 | Olomouc (Czech Republic) | 14.14 |  | 1 | 0 | 49.55 | 12 |
| Acrocephalus_palustris2 | Olomouc (Czech Republic) | 7.07 |  | 1 | 1 | 49.55 | 12 |
| Acrocephalus_palustris3 | Brønderslev (Denmark) | 8.76 | 2.2 | 10 | 0 | 57.2 | 12 |
| Acrocephalus_schoenobaenus | Brønderslev (Denmark) | 6.93 | 3.08 | 41 | 0 | 57.2 | 11.9 |
| Acrocephalus_scirpaceus1 | Brønderslev (Denmark) | 6.59 | 2.7 | 6 | 0 | 57.2 | 11.8 |
| Acrocephalus_scirpaceus2 | Paris (France) | 3.16 |  | 2 | 1 | 48.1 | 11.8 |
| Actitis_hypoleucos1 | Brønderslev (Denmark) | 17.5 | 0.71 | 2 | 0 | 57.2 | 47.8 |
| Actitis_hypoleucos2 | Brønderslev (Denmark) | 12 |  | 1 | 1 | 57.2 | 47.8 |
| Actitis_hypoleucos3 | Paris (France) | 20 | 1 | 1 | 0 | 48.1 | 47.8 |
| Aegithalos_caudatus1 | Paris (France) | 5.27 | 0.97 | 11 | 0 | 48.1 | 8.8 |
| Aegithalos_caudatus2 | Paris (France) | 4.58 | 0.76 | 6 | 1 | 48.1 | 8.8 |
| Aegithalos_caudatus3 | Granada (Spain) | 7.2 | 6.78 | 5 | 0 | 37.25 | 8.8 |
| Aegithalos_caudatus4 | Budapest (Hungary) | 6.36 | 1.84 | 4 | 0 | 47.45 | 8.8 |
| Aegithalos_caudatus5 | Budapest (Hungary) | 4.55 | 1.75 | 13 | 1 | 47.45 | 8.8 |
| Aegithalos_caudatus6 | Toledo (Spain) | 18.97 |  | 1 | 0 | 39.83 | 8.8 |
| Aix_galericulata | Toledo (Spain) | 20 |  | 1 | 0 | 39.83 | 560 |
| Alauda_arvensis1 | Brønderslev (Denmark) | 31.42 | 28.17 | 43 | 0 | 57.2 | 36.4 |
| Alauda_arvensis2 | Poznan (Poland) | 45.44 | 35.1 | 2 | 0 | 52.45 | 36.4 |
| Alca_torda | Oslo (Norway) | 70 |  | 1 | 1 | 59.92 | 722 |
| Alcedo_atthis | Paris (France) | 8.54 |  | 1 | 0 | 48.1 | 32.4 |
| Alectoris_rufa1 | Granada (Spain) | 30 | 13.9 | 6 | 0 | 37.25 | 477.5 |
| Alectoris_rufa2 | Toledo (Spain) | 42 | 25.15 | 5 | 0 | 39.83 | 477.5 |
| Alectoris_rufa3 | Toledo (Spain) | 30 |  | 1 | 1 | 39.83 | 477.5 |
| Anas_crecca | Brønderslev (Denmark) | 39.23 | 12.05 | 22 | 0 | 57.2 | 286.5 |
| Anas_platyrhynchos1 | Olomouc (Czech Republic) | 1.31 | 1.7 | 13 | 1 | 49.55 | 1119 |
| Anas_platyrhynchos10 | Poznan (Poland) | 88 |  | 1 | 0 | 52.45 | 1119 |
| Anas_platyrhynchos11 | Poznan (Poland) | 3 | 0.89 | 6 | 1 | 52.45 | 1119 |
| Anas_platyrhynchos12 | Toledo (Spain) | 15 |  | 1 | 1 | 39.83 | 1119 |
| Anas_platyrhynchos2 | Brønderslev (Denmark) | 28.76 | 16.62 | 34 | 0 | 57.2 | 1119 |
| Anas_platyrhynchos3 | Brønderslev (Denmark) | 5 | 2.05 | 11 | 1 | 57.2 | 1119 |
| Anas_platyrhynchos4 | Paris (France) | 4.81 | 1.52 | 26 | 0 | 48.1 | 1119 |
| Anas_platyrhynchos5 | Paris (France) | 2.95 | 1.22 | 41 | 1 | 48.1 | 1119 |
| Anas_platyrhynchos6 | Granada (Spain) | 5.71 | 4.5 | 7 | 1 | 37.25 | 1119 |
| Anas_platyrhynchos7 | Budapest (Hungary) | 3.75 | 0.96 | 4 | 1 | 47.45 | 1119 |
| Anas_platyrhynchos8 | Oslo (Norway) | 11.11 | 3.76 | 18 | 0 | 59.92 | 1119 |
| Anas_platyrhynchos9 | Oslo (Norway) | 4.96 | 1.78 | 52 | 1 | 59.92 | 1119 |
| Anser_anser1 | Brønderslev (Denmark) | 180 |  | 4 | 0 | 57.2 | 3464.5 |
| Anser_anser2 | Oslo (Norway) | 12.42 | 3.99 | 24 | 1 | 59.92 | 3464.5 |
| Anthus_campestris | Brønderslev (Denmark) | 18.2 |  | 2 | 0 | 57.2 | 28.8 |
| Anthus_pratensis1 | Brønderslev (Denmark) | 13.22 | 6.62 | 55 | 0 | 57.2 | 19.3 |
| Anthus_pratensis2 | Rovaniemi (Finland) | 28.31 | 10.88 | 2 | 0 | 66.45 | 19.3 |
| Anthus_pratensis3 | Oslo (Norway) | 5.82 | 3.54 | 54 | 0 | 59.92 | 19.3 |
| Anthus_pratensis4 | Poznan (Poland) | 32 |  | 1 | 0 | 52.45 | 19.3 |
| Anthus_spinoletta1 | Paris (France) | 7.5 | 4.32 | 6 | 0 | 48.1 | 21.5 |
| Anthus_spinoletta2 | Paris (France) | 3.05 | 1.24 | 18 | 1 | 48.1 | 21.5 |
| Anthus_trivialis | Brønderslev (Denmark) | 10.9 | 4.77 | 35 | 0 | 57.2 | 23.4 |
| Apus_apus | Brønderslev (Denmark) | 38.1 | 0.52 | 2 | 0 | 57.2 | 39.7 |
| Ardea_cinerea1 | Brønderslev (Denmark) | 62.08 | 45.08 | 13 | 0 | 57.2 | 1433 |
| Ardea_cinerea2 | Paris (France) | 26 | 16.37 | 3 | 0 | 48.1 | 1433 |
| Ardea_cinerea3 | Paris (France) | 20.55 | 12.14 | 11 | 1 | 48.1 | 1433 |
| Ardea_cinerea4 | Oslo (Norway) | 50 |  | 1 | 0 | 59.92 | 1433 |
| Athene_noctua1 | Granada (Spain) | 46.3 | 36.93 | 11 | 0 | 37.25 | 168 |
| Athene_noctua2 | Toledo (Spain) | 20.22 |  | 1 | 0 | 39.83 | 168 |
| Aythya_fuligula1 | Brønderslev (Denmark) | 10.68 | 2.34 | 34 | 0 | 57.2 | 656.5 |
| Aythya_fuligula2 | Oslo (Norway) | 9 |  | 1 | 1 | 59.92 | 656.5 |
| Branta_leucopsis | Oslo (Norway) | 12.55 | 8.72 | 4 | 0 | 59.92 | 1630.5 |
| Bubulcus_ibis | Granada (Spain) | 65 |  | 1 | 0 | 37.25 | 307.5 |
| Bucephala_clangula1 | Oslo (Norway) | 18 |  | 1 | 0 | 59.92 | 840.3 |
| Bucephala_clangula2 | Oslo (Norway) | 10.4 | 6.66 | 5 | 1 | 59.92 | 840.3 |
| Buteo_buteo1 | Olomouc (Czech Republic) | 55.26 | 15.07 | 3 | 0 | 49.55 | 806.5 |
| Buteo_buteo2 | Brønderslev (Denmark) | 60.01 | 22.8 | 15 | 0 | 57.2 | 806.5 |
| Caprimulgus_ruficollis | Granada (Spain) | 24 |  | 1 | 0 | 37.25 | 85 |
| Carduelis_cannabina1 | Olomouc (Czech Republic) | 14.93 | 5.73 | 4 | 0 | 49.55 | 19 |
| Carduelis_cannabina2 | Brønderslev (Denmark) | 10.8 | 4.71 | 54 | 0 | 57.2 | 19 |
| Carduelis_cannabina3 | Poznan (Poland) | 12.89 | 7.06 | 13 | 0 | 52.45 | 19 |
| Carduelis_cannabina4 | Toledo (Spain) | 18.51 | 8.48 | 6 | 0 | 39.83 | 19 |
| Carduelis_cannabina5 | Toledo (Spain) | 9.19 | 4.39 | 13 | 1 | 39.83 | 19 |
| Carduelis_carduelis1 | Olomouc (Czech Republic) | 14.95 | 8.22 | 7 | 0 | 49.55 | 15.6 |
| Carduelis_carduelis10 | Budapest (Hungary) | 7.89 | 2.24 | 5 | 1 | 47.45 | 15.6 |
| Carduelis_carduelis11 | Oslo (Norway) | 4.95 | 1.53 | 15 | 0 | 59.92 | 15.6 |
| Carduelis_carduelis12 | Oslo (Norway) | 17.69 |  | 1 | 1 | 59.92 | 15.6 |
| Carduelis_carduelis13 | Toledo (Spain) | 16.33 | 8.62 | 22 | 0 | 39.83 | 15.6 |
| Carduelis_carduelis14 | Toledo (Spain) | 12.4 | 5.73 | 20 | 1 | 39.83 | 15.6 |
| Carduelis_carduelis2 | Olomouc (Czech Republic) | 3.61 |  | 1 | 1 | 49.55 | 15.6 |
| Carduelis_carduelis3 | Brønderslev (Denmark) | 10.77 | 3.76 | 4 | 0 | 57.2 | 15.6 |
| Carduelis_carduelis4 | Brønderslev (Denmark) | 3 |  | 2 | 1 | 57.2 | 15.6 |
| Carduelis_carduelis5 | Paris (France) | 11 |  | 2 | 0 | 48.1 | 15.6 |
| Carduelis_carduelis6 | Paris (France) | 5.86 | 1.21 | 7 | 1 | 48.1 | 15.6 |
| Carduelis_carduelis7 | Granada (Spain) | 20.63 | 11.9 | 90 | 0 | 37.25 | 15.6 |
| Carduelis_carduelis8 | Granada (Spain) | 9.1 | 6.2 | 50 | 1 | 37.25 | 15.6 |
| Carduelis_carduelis9 | Budapest (Hungary) | 8.99 | 5.96 | 9 | 0 | 47.45 | 15.6 |
| Carduelis_flammea1 | Brønderslev (Denmark) | 4.5 | 0.71 | 2 | 0 | 57.2 | 13.1 |
| Carduelis_flammea2 | Oslo (Norway) | 12 |  | 2 | 0 | 59.92 | 13.1 |
| Carduelis_flammea3 | Oslo (Norway) | 6 |  | 2 | 1 | 59.92 | 13.1 |
| Carduelis_flavirostris | Oslo (Norway) | 8 |  | 2 | 0 | 59.92 | 16.1 |
| Carduelis_spinus1 | Brønderslev (Denmark) | 3.91 |  | 1 | 0 | 57.2 | 13.8 |
| Carduelis_spinus2 | Rovaniemi (Finland) | 10.11 | 3.84 | 3 | 0 | 66.45 | 13.8 |
| Carduelis_spinus3 | Paris (France) | 4.92 | 0.84 | 12 | 0 | 48.1 | 13.8 |
| Carpodacus_erythrinus | Rovaniemi (Finland) | 9.44 | 4.75 | 2 | 0 | 66.45 | 13.8 |
| Certhia_brachydactyla1 | Paris (France) | 7.12 | 1.22 | 4 | 0 | 48.1 | 9.2 |
| Certhia_brachydactyla2 | Paris (France) | 7.11 | 2.13 | 9 | 1 | 48.1 | 9.2 |
| Certhia_brachydactyla3 | Toledo (Spain) | 11.15 | 2.59 | 5 | 0 | 39.83 | 9.2 |
| Certhia_brachydactyla4 | Toledo (Spain) | 7.35 | 1.31 | 4 | 1 | 39.83 | 9.2 |
| Certhia_familiaris1 | Brønderslev (Denmark) | 4.47 |  | 2 | 0 | 57.2 | 9.2 |
| Certhia_familiaris2 | Oslo (Norway) | 7.23 | 1.98 | 4 | 0 | 59.92 | 9.2 |
| Cettia_cetti1 | Granada (Spain) | 50.01 |  | 1 | 0 | 37.25 | 14.1 |
| Cettia_cetti2 | Toledo (Spain) | 10.05 |  | 1 | 0 | 39.83 | 14.1 |
| Charadrius_hiaticula | Brønderslev (Denmark) | 20.45 | 7.19 | 22 | 0 | 57.2 | 63.3 |
| Chloris_chloris1 | Olomouc (Czech Republic) | 12.98 | 5.34 | 12 | 0 | 49.55 | 27.7 |
| Chloris_chloris10 | Granada (Spain) | 11.61 | 5.81 | 94 | 1 | 37.25 | 27.7 |
| Chloris_chloris11 | Budapest (Hungary) | 18.43 | 12.81 | 3 | 0 | 47.45 | 27.7 |
| Chloris_chloris12 | Budapest (Hungary) | 8.87 | 4.5 | 10 | 1 | 47.45 | 27.7 |
| Chloris_chloris13 | Oslo (Norway) | 5.88 | 1.3 | 10 | 0 | 59.92 | 27.7 |
| Chloris_chloris14 | Oslo (Norway) | 9.17 | 3.27 | 19 | 1 | 59.92 | 27.7 |
| Chloris_chloris15 | Poznan (Poland) | 15.11 | 8.33 | 2 | 0 | 52.45 | 27.7 |
| Chloris_chloris16 | Toledo (Spain) | 17.72 | 5.68 | 18 | 0 | 39.83 | 27.7 |
| Chloris_chloris17 | Toledo (Spain) | 11.56 | 4.53 | 20 | 1 | 39.83 | 27.7 |
| Chloris_chloris2 | Olomouc (Czech Republic) | 6.31 | 2.09 | 10 | 1 | 49.55 | 27.7 |
| Chloris_chloris3 | Brønderslev (Denmark) | 6.53 | 2.45 | 29 | 0 | 57.2 | 27.7 |
| Chloris_chloris4 | Brønderslev (Denmark) | 6.8 | 1.48 | 9 | 1 | 57.2 | 27.7 |
| Chloris_chloris5 | Rovaniemi (Finland) | 15.22 | 12.31 | 9 | 0 | 66.45 | 27.7 |
| Chloris_chloris6 | Rovaniemi (Finland) | 13.35 | 5.02 | 9 | 1 | 66.45 | 27.7 |
| Chloris_chloris7 | Paris (France) | 7.65 | 3.25 | 21 | 0 | 48.1 | 27.7 |
| Chloris_chloris8 | Paris (France) | 5.32 | 2.13 | 16 | 1 | 48.1 | 27.7 |
| Chloris_chloris9 | Granada (Spain) | 23.46 | 11.37 | 22 | 0 | 37.25 | 27.7 |
| Cinclus_cinclus | Oslo (Norway) | 13 | 1.41 | 2 | 0 | 59.92 | 61.9 |
| Cisticola_juncidis1 | Granada (Spain) | 25.05 | 31.57 | 4 | 0 | 37.25 | 8.5 |
| Cisticola_juncidis2 | Toledo (Spain) | 15.03 |  | 1 | 0 | 39.83 | 8.5 |
| Clamator_glandarius | Granada (Spain) | 55.6 | 15.17 | 7 | 0 | 37.25 | 153.5 |
| Coccothraustes_coccothraustes1 | Olomouc (Czech Republic) | 22.17 | 10.83 | 5 | 0 | 49.55 | 54.7 |
| Coccothraustes_coccothraustes2 | Olomouc (Czech Republic) | 15 | 10.15 | 3 | 1 | 49.55 | 54.7 |
| Coccothraustes_coccothraustes3 | Paris (France) | 5.1 |  | 1 | 0 | 48.1 | 54.7 |
| Coccothraustes_coccothraustes4 | Paris (France) | 8.6 |  | 1 | 1 | 48.1 | 54.7 |
| Coccothraustes_coccothraustes5 | Granada (Spain) | 25.44 | 9.82 | 5 | 0 | 37.25 | 54.7 |
| Coccothraustes_coccothraustes6 | Budapest (Hungary) | 24 |  | 1 | 0 | 47.45 | 54.7 |
| Coccothraustes_coccothraustes7 | Poznan (Poland) | 8.54 |  | 2 | 1 | 52.45 | 54.7 |
| Coccothraustes_coccothraustes8 | Toledo (Spain) | 8.49 |  | 1 | 1 | 39.83 | 54.7 |
| Columba_livia1 | Brønderslev (Denmark) | 28 | 19.8 | 2 | 0 | 57.2 | 261 |
| Columba_livia10 | Oslo (Norway) | 4.05 | 1.61 | 19 | 1 | 59.92 | 261 |
| Columba_livia11 | Toledo (Spain) | 24.81 | 10.82 | 6 | 0 | 39.83 | 261 |
| Columba_livia12 | Toledo (Spain) | 15.83 | 5.7 | 11 | 1 | 39.83 | 261 |
| Columba_livia2 | Rovaniemi (Finland) | 5.67 | 3.75 | 26 | 1 | 66.45 | 261 |
| Columba_livia3 | Paris (France) | 8 |  | 2 | 0 | 48.1 | 261 |
| Columba_livia4 | Paris (France) | 2.55 | 0.52 | 11 | 1 | 48.1 | 261 |
| Columba_livia5 | Granada (Spain) | 78.61 | 40.61 | 143 | 0 | 37.25 | 261 |
| Columba_livia6 | Granada (Spain) | 3.61 | 2.63 | 248 | 1 | 37.25 | 261 |
| Columba_livia7 | Budapest (Hungary) | 6 | 5.96 | 6 | 0 | 47.45 | 261 |
| Columba_livia8 | Budapest (Hungary) | 5.81 | 3.07 | 90 | 1 | 47.45 | 261 |
| Columba_livia9 | Oslo (Norway) | 8 |  | 1 | 0 | 59.92 | 261 |
| Columba_oenas1 | Paris (France) | 17.75 | 4.35 | 4 | 0 | 48.1 | 494.5 |
| Columba_oenas2 | Paris (France) | 5 |  | 1 | 1 | 48.1 | 494.5 |
| Columba_oenas3 | Oslo (Norway) | 15.27 | 9.51 | 2 | 0 | 59.92 | 494.5 |
| Columba_oenas4 | Oslo (Norway) | 8.94 |  | 1 | 1 | 59.92 | 494.5 |
| Columba_palumbus1 | Olomouc (Czech Republic) | 27.26 | 11.56 | 5 | 0 | 49.55 | 494.5 |
| Columba_palumbus10 | Budapest (Hungary) | 21.47 | 11.48 | 21 | 0 | 47.45 | 494.5 |
| Columba_palumbus11 | Budapest (Hungary) | 7.5 | 3 | 4 | 1 | 47.45 | 494.5 |
| Columba_palumbus12 | Oslo (Norway) | 7 | 3.97 | 25 | 0 | 59.92 | 494.5 |
| Columba_palumbus13 | Oslo (Norway) | 6.23 | 3.12 | 54 | 1 | 59.92 | 494.5 |
| Columba_palumbus14 | Poznan (Poland) | 60.41 |  | 1 | 0 | 52.45 | 494.5 |
| Columba_palumbus15 | Poznan (Poland) | 6.66 | 5.99 | 19 | 1 | 52.45 | 494.5 |
| Columba_palumbus16 | Toledo (Spain) | 26.77 | 16.49 | 33 | 0 | 39.83 | 494.5 |
| Columba_palumbus17 | Toledo (Spain) | 15.52 | 9.72 | 13 | 1 | 39.83 | 494.5 |
| Columba_palumbus2 | Olomouc (Czech Republic) | 8.22 | 3.81 | 70 | 1 | 49.55 | 494.5 |
| Columba_palumbus3 | Brønderslev (Denmark) | 28.17 | 16.16 | 97 | 0 | 57.2 | 494.5 |
| Columba_palumbus4 | Brønderslev (Denmark) | 9.55 | 7.78 | 39 | 1 | 57.2 | 494.5 |
| Columba_palumbus5 | Rovaniemi (Finland) | 30 |  | 1 | 0 | 66.45 | 494.5 |
| Columba_palumbus6 | Paris (France) | 14.43 | 7.96 | 45 | 0 | 48.1 | 494.5 |
| Columba_palumbus7 | Paris (France) | 8.81 | 3.63 | 36 | 1 | 48.1 | 494.5 |
| Columba_palumbus8 | Granada (Spain) | 107.72 | 54.37 | 118 | 0 | 37.25 | 494.5 |
| Columba_palumbus9 | Granada (Spain) | 10.75 | 8.9 | 11 | 1 | 37.25 | 494.5 |
| Corvus_corax | Brønderslev (Denmark) | 78.06 | 13.18 | 13 | 0 | 57.2 | 1200.6 |
| Corvus_cornix1 | Olomouc (Czech Republic) | 13.89 |  | 1 | 0 | 49.55 | 544.5 |
| Corvus_cornix10 | Poznan (Poland) | 6.38 | 3.16 | 8 | 1 | 52.45 | 544.5 |
| Corvus_cornix2 | Brønderslev (Denmark) | 41.15 | 17.18 | 73 | 0 | 57.2 | 544.5 |
| Corvus_cornix3 | Brønderslev (Denmark) | 11.38 | 6.77 | 7 | 1 | 57.2 | 544.5 |
| Corvus_cornix4 | Rovaniemi (Finland) | 31.69 | 4.59 | 2 | 0 | 66.45 | 544.5 |
| Corvus_cornix5 | Rovaniemi (Finland) | 20.63 | 14.25 | 17 | 1 | 66.45 | 544.5 |
| Corvus_cornix6 | Budapest (Hungary) | 24.09 | 10.66 | 2 | 0 | 47.45 | 544.5 |
| Corvus_cornix7 | Budapest (Hungary) | 14.44 | 6 | 35 | 1 | 47.45 | 544.5 |
| Corvus_cornix8 | Oslo (Norway) | 17.3 | 5.42 | 21 | 0 | 59.92 | 544.5 |
| Corvus_cornix9 | Oslo (Norway) | 7.5 | 3.2 | 49 | 1 | 59.92 | 544.5 |
| Corvus_corone1 | Paris (France) | 20.41 | 11.09 | 35 | 0 | 48.1 | 544.5 |
| Corvus_corone2 | Paris (France) | 15.71 | 9.58 | 32 | 1 | 48.1 | 544.5 |
| Corvus_corone3 | Poznan (Poland) | 4.5 | 2.12 | 2 | 1 | 52.45 | 544.5 |
| Corvus_frugilegus1 | Brønderslev (Denmark) | 46.53 | 11.73 | 99 | 0 | 57.2 | 453.5 |
| Corvus_frugilegus2 | Brønderslev (Denmark) | 19.26 | 8.59 | 24 | 1 | 57.2 | 453.5 |
| Corvus_frugilegus3 | Poznan (Poland) | 5.5 | 4.32 | 5 | 1 | 52.45 | 453.5 |
| Corvus_monedula1 | Olomouc (Czech Republic) | 7 | 4.9 | 4 | 1 | 49.55 | 249 |
| Corvus_monedula10 | Toledo (Spain) | 31.04 | 9.98 | 5 | 0 | 39.83 | 249 |
| Corvus_monedula11 | Toledo (Spain) | 21.73 | 8.69 | 13 | 1 | 39.83 | 249 |
| Corvus_monedula2 | Brønderslev (Denmark) | 32.11 | 10.96 | 45 | 0 | 57.2 | 249 |
| Corvus_monedula3 | Brønderslev (Denmark) | 9.92 | 5.28 | 26 | 1 | 57.2 | 249 |
| Corvus_monedula4 | Rovaniemi (Finland) | 60 |  | 1 | 0 | 66.45 | 249 |
| Corvus_monedula5 | Granada (Spain) | 113.42 | 52.64 | 30 | 0 | 37.25 | 249 |
| Corvus_monedula6 | Oslo (Norway) | 8.92 | 4.36 | 8 | 0 | 59.92 | 249 |
| Corvus_monedula7 | Oslo (Norway) | 7.78 | 3.53 | 19 | 1 | 59.92 | 249 |
| Corvus_monedula8 | Poznan (Poland) | 18.23 | 0.29 | 2 | 0 | 52.45 | 249 |
| Corvus_monedula9 | Poznan (Poland) | 3.34 | 2.71 | 14 | 1 | 52.45 | 249 |
| Cuculus_canorus1 | Brønderslev (Denmark) | 21.72 | 12.97 | 13 | 0 | 57.2 | 120.5 |
| Cuculus_canorus2 | Brønderslev (Denmark) | 33.5 | 2.12 | 2 | 1 | 57.2 | 120.5 |
| Cyanopica_cyana | Granada (Spain) | 56.86 | 23.36 | 46 | 0 | 37.25 | 71 |
| Cygnus_olor1 | Oslo (Norway) | 4.47 |  | 2 | 0 | 59.92 | 10750 |
| Cygnus_olor2 | Oslo (Norway) | 1 |  | 1 | 1 | 59.92 | 10750 |
| Delichon_urbica1 | Paris (France) | 6.35 | 0.45 | 27 | 1 | 48.1 | 19.6 |
| Delichon_urbica2 | Granada (Spain) | 32 | 10.21 | 16 | 1 | 37.25 | 19.6 |
| Delichon_urbica3 | Toledo (Spain) | 35.51 |  | 1 | 0 | 39.83 | 19.6 |
| Delichon_urbica4 | Toledo (Spain) | 8.67 | 1.15 | 3 | 1 | 39.83 | 19.6 |
| Dendrocopos_major1 | Olomouc (Czech Republic) | 18.56 | 8.17 | 6 | 0 | 49.55 | 89.7 |
| Dendrocopos_major10 | Oslo (Norway) | 9.85 |  | 1 | 0 | 59.92 | 89.7 |
| Dendrocopos_major11 | Toledo (Spain) | 10.77 |  | 1 | 1 | 39.83 | 89.7 |
| Dendrocopos_major2 | Olomouc (Czech Republic) | 9.43 | 4.37 | 4 | 1 | 49.55 | 89.7 |
| Dendrocopos_major3 | Brønderslev (Denmark) | 14.2 | 7.22 | 9 | 0 | 57.2 | 89.7 |
| Dendrocopos_major4 | Rovaniemi (Finland) | 12 |  | 1 | 1 | 66.45 | 89.7 |
| Dendrocopos_major5 | Paris (France) | 14 |  | 1 | 0 | 48.1 | 89.7 |
| Dendrocopos_major6 | Paris (France) | 9.37 | 1.69 | 3 | 1 | 48.1 | 89.7 |
| Dendrocopos_major7 | Granada (Spain) | 58.14 |  | 1 | 0 | 37.25 | 89.7 |
| Dendrocopos_major8 | Budapest (Hungary) | 32.98 |  | 1 | 0 | 47.45 | 89.7 |
| Dendrocopos_major9 | Budapest (Hungary) | 7.17 | 0.67 | 5 | 1 | 47.45 | 89.7 |
| Dryocopus_martius1 | Paris (France) | 38.71 | 17.97 | 2 | 0 | 48.1 | 273 |
| Dryocopus_martius2 | Oslo (Norway) | 10 |  | 1 | 0 | 59.92 | 273 |
| Dryocopus_martius3 | Poznan (Poland) | 50.16 |  | 1 | 0 | 52.45 | 273 |
| Egretta_garzetta | Paris (France) | 24.5 | 4.95 | 2 | 0 | 48.1 | 532.5 |
| Emberiza_cirlus | Toledo (Spain) | 15 |  | 1 | 0 | 39.83 | 23.8 |
| Emberiza_citrinella1 | Olomouc (Czech Republic) | 15.54 | 7.33 | 37 | 0 | 49.55 | 26.8 |
| Emberiza_citrinella2 | Olomouc (Czech Republic) | 8 |  | 1 | 1 | 49.55 | 26.8 |
| Emberiza_citrinella3 | Brønderslev (Denmark) | 9.79 | 5.3 | 107 | 0 | 57.2 | 26.8 |
| Emberiza_citrinella4 | Rovaniemi (Finland) | 10.43 | 5.65 | 3 | 0 | 66.45 | 26.8 |
| Emberiza_citrinella5 | Rovaniemi (Finland) | 6.73 | 3 | 3 | 1 | 66.45 | 26.8 |
| Emberiza_citrinella6 | Oslo (Norway) | 8.2 | 2.17 | 5 | 0 | 59.92 | 26.8 |
| Emberiza_citrinella7 | Oslo (Norway) | 14 |  | 1 | 1 | 59.92 | 26.8 |
| Emberiza_citrinella8 | Poznan (Poland) | 3.08 | 0.11 | 2 | 0 | 52.45 | 26.8 |
| Emberiza_schoeniclus1 | Olomouc (Czech Republic) | 28.07 |  | 1 | 0 | 49.55 | 18.8 |
| Emberiza_schoeniclus2 | Brønderslev (Denmark) | 9.17 | 4.8 | 76 | 0 | 57.2 | 18.8 |
| Emberiza_schoeniclus3 | Brønderslev (Denmark) | 8.73 | 0.69 | 2 | 1 | 57.2 | 18.8 |
| Emberiza_schoeniclus4 | Rovaniemi (Finland) | 8 |  | 1 | 1 | 66.45 | 18.8 |
| Emberiza_schoeniclus5 | Oslo (Norway) | 8 | 2.14 | 11 | 0 | 59.92 | 18.8 |
| Emberiza_schoeniclus6 | Poznan (Poland) | 36.51 | 4.95 | 2 | 0 | 52.45 | 18.8 |
| Erithacus_rubecula1 | Oslo (Norway) | 6.06 | 4.51 | 9 | 1 | 59.92 | 16.4 |
| Erithacus_rubecula10 | Oslo (Norway) | 5.47 | 3.53 | 18 | 0 | 59.92 | 16.4 |
| Erithacus_rubecula11 | Poznan (Poland) | 8 |  | 1 | 1 | 52.45 | 16.4 |
| Erithacus_rubecula12 | Toledo (Spain) | 2.24 |  | 1 | 0 | 39.83 | 16.4 |
| Erithacus_rubecula2 | Olomouc (Czech Republic) | 16.71 | 5.63 | 10 | 0 | 49.55 | 16.4 |
| Erithacus_rubecula3 | Olomouc (Czech Republic) | 3.42 | 1.85 | 4 | 1 | 49.55 | 16.4 |
| Erithacus_rubecula4 | Brønderslev (Denmark) | 8.91 | 3.19 | 10 | 0 | 57.2 | 16.4 |
| Erithacus_rubecula5 | Brønderslev (Denmark) | 5.47 | 2.16 | 3 | 1 | 57.2 | 16.4 |
| Erithacus_rubecula6 | Paris (France) | 5.19 | 1.71 | 50 | 0 | 48.1 | 16.4 |
| Erithacus_rubecula7 | Paris (France) | 4.38 | 2.07 | 65 | 1 | 48.1 | 16.4 |
| Erithacus_rubecula8 | Granada (Spain) | 10.15 | 5.88 | 5 | 1 | 37.25 | 16.4 |
| Erithacus_rubecula9 | Budapest (Hungary) | 13.05 | 8.56 | 2 | 1 | 47.45 | 16.4 |
| Falco_tinnunculus1 | Olomouc (Czech Republic) | 50.58 | 13.71 | 3 | 0 | 49.55 | 174.5 |
| Falco_tinnunculus2 | Brønderslev (Denmark) | 28.35 | 17.19 | 6 | 0 | 57.2 | 174.5 |
| Falco_tinnunculus3 | Paris (France) | 6.32 |  | 1 | 0 | 48.1 | 174.5 |
| Falco_tinnunculus4 | Granada (Spain) | 115.49 | 29.26 | 5 | 0 | 37.25 | 174.5 |
| Falco_tinnunculus5 | Budapest (Hungary) | 25 |  | 1 | 0 | 47.45 | 174.5 |
| Ficedula_hypoleuca1 | Brønderslev (Denmark) | 5.39 |  | 1 | 0 | 57.2 | 14.4 |
| Ficedula_hypoleuca2 | Rovaniemi (Finland) | 6.59 | 2.17 | 4 | 0 | 66.45 | 14.4 |
| Ficedula_hypoleuca3 | Rovaniemi (Finland) | 7.75 | 0.66 | 3 | 1 | 66.45 | 14.4 |
| Ficedula_hypoleuca4 | Paris (France) | 5.39 |  | 1 | 1 | 48.1 | 14.4 |
| Ficedula_hypoleuca5 | Budapest (Hungary) | 6.71 |  | 1 | 0 | 47.45 | 14.4 |
| Ficedula_hypoleuca6 | Oslo (Norway) | 7.07 |  | 1 | 0 | 59.92 | 14.4 |
| Fringilla_coelebs1 | Olomouc (Czech Republic) | 13.62 | 3.96 | 8 | 0 | 49.55 | 24.2 |
| Fringilla_coelebs10 | Budapest (Hungary) | 7.23 | 4.38 | 11 | 1 | 47.45 | 24.2 |
| Fringilla_coelebs11 | Oslo (Norway) | 7.3 | 3.77 | 31 | 0 | 59.92 | 24.2 |
| Fringilla_coelebs12 | Oslo (Norway) | 4.43 | 1.16 | 21 | 1 | 59.92 | 24.2 |
| Fringilla_coelebs13 | Poznan (Poland) | 8.76 | 2.38 | 2 | 0 | 52.45 | 24.2 |
| Fringilla_coelebs14 | Poznan (Poland) | 4.93 | 3.34 | 5 | 1 | 52.45 | 24.2 |
| Fringilla_coelebs15 | Toledo (Spain) | 36.9 |  | 1 | 0 | 39.83 | 24.2 |
| Fringilla_coelebs2 | Olomouc (Czech Republic) | 4.57 | 4.77 | 29 | 1 | 49.55 | 24.2 |
| Fringilla_coelebs3 | Brønderslev (Denmark) | 9.39 | 4.59 | 103 | 0 | 57.2 | 24.2 |
| Fringilla_coelebs4 | Brønderslev (Denmark) | 5.6 | 1.93 | 18 | 1 | 57.2 | 24.2 |
| Fringilla_coelebs5 | Rovaniemi (Finland) | 8.5 | 2.12 | 2 | 0 | 66.45 | 24.2 |
| Fringilla_coelebs6 | Rovaniemi (Finland) | 6.76 | 3.8 | 17 | 1 | 66.45 | 24.2 |
| Fringilla_coelebs7 | Paris (France) | 6.55 | 2.74 | 34 | 0 | 48.1 | 24.2 |
| Fringilla_coelebs8 | Paris (France) | 5.94 | 1.36 | 24 | 1 | 48.1 | 24.2 |
| Fringilla_coelebs9 | Granada (Spain) | 35.71 | 14.6 | 24 | 0 | 37.25 | 24.2 |
| Fulica_atra | Brønderslev (Denmark) | 19.87 | 8.82 | 15 | 0 | 57.2 | 732.5 |
| Galerida_cristata1 | Granada (Spain) | 72.84 | 32.59 | 36 | 0 | 37.25 | 44.7 |
| Galerida_cristata2 | Poznan (Poland) | 2 |  | 1 | 1 | 52.45 | 44.7 |
| Galerida_cristata3 | Toledo (Spain) | 10.53 | 9.8 | 4 | 0 | 39.83 | 44.7 |
| Galerida_cristata4 | Toledo (Spain) | 17.18 | 7.68 | 12 | 1 | 39.83 | 44.7 |
| Gallinago_gallinago1 | Brønderslev (Denmark) | 25.83 | 25.28 | 6 | 0 | 57.2 | 106.5 |
| Gallinago_gallinago2 | Rovaniemi (Finland) | 54.15 |  | 1 | 0 | 66.45 | 106.5 |
| Gallinula_chloropus1 | Brønderslev (Denmark) | 20 |  | 1 | 0 | 57.2 | 348.5 |
| Gallinula_chloropus2 | Brønderslev (Denmark) | 6.5 | 0.71 | 2 | 1 | 57.2 | 348.5 |
| Gallinula_chloropus3 | Paris (France) | 10.32 | 5.58 | 19 | 0 | 48.1 | 348.5 |
| Gallinula_chloropus4 | Paris (France) | 5.92 | 2.47 | 32 | 1 | 48.1 | 348.5 |
| Gallinula_chloropus5 | Oslo (Norway) | 8 |  | 1 | 0 | 59.92 | 348.5 |
| Gallinula_chloropus6 | Oslo (Norway) | 14 |  | 1 | 1 | 59.92 | 348.5 |
| Garrulus_glandarius1 | Olomouc (Czech Republic) | 39.15 | 23.01 | 10 | 0 | 49.55 | 161.7 |
| Garrulus_glandarius2 | Olomouc (Czech Republic) | 7.05 | 7.7 | 5 | 1 | 49.55 | 161.7 |
| Garrulus_glandarius3 | Brønderslev (Denmark) | 20.95 | 9.01 | 16 | 0 | 57.2 | 161.7 |
| Garrulus_glandarius4 | Paris (France) | 11.16 | 4.83 | 12 | 0 | 48.1 | 161.7 |
| Garrulus_glandarius5 | Paris (France) | 7.44 | 2.73 | 14 | 1 | 48.1 | 161.7 |
| Garrulus_glandarius6 | Budapest (Hungary) | 27.87 | 3.6 | 2 | 0 | 47.45 | 161.7 |
| Garrulus_glandarius7 | Budapest (Hungary) | 7.54 | 4.02 | 4 | 1 | 47.45 | 161.7 |
| Garrulus_glandarius8 | Poznan (Poland) | 6.87 | 3.78 | 4 | 1 | 52.45 | 161.7 |
| Grus_grus1 | Brønderslev (Denmark) | 108 |  | 1 | 0 | 57.2 | 4541.5 |
| Grus_grus2 | Poznan (Poland) | 100 |  | 1 | 0 | 52.45 | 4541.5 |
| Haematopus_ostralegus1 | Brønderslev (Denmark) | 40.01 |  | 1 | 0 | 57.2 | 531 |
| Haematopus_ostralegus2 | Oslo (Norway) | 19 | 6.77 | 14 | 0 | 59.92 | 531 |
| Haematopus_ostralegus3 | Oslo (Norway) | 28 |  | 3 | 1 | 59.92 | 531 |
| Hippolais_icterina1 | Brønderslev (Denmark) | 7.76 | 3.2 | 17 | 0 | 57.2 | 13.3 |
| Hippolais_icterina2 | Brønderslev (Denmark) | 7.69 | 2.67 | 4 | 1 | 57.2 | 13.3 |
| Hippolais_polyglotta1 | Granada (Spain) | 15.95 | 4 | 5 | 0 | 37.25 | 11.5 |
| Hippolais_polyglotta2 | Toledo (Spain) | 18.14 | 11.03 | 3 | 0 | 39.83 | 11.5 |
| Hirundo_daurica | Toledo (Spain) | 6 |  | 1 | 1 | 39.83 | 22.3 |
| Hirundo_rustica1 | Olomouc (Czech Republic) | 15.63 | 5.16 | 3 | 0 | 49.55 | 19.1 |
| Hirundo_rustica2 | Brønderslev (Denmark) | 10.15 | 4.98 | 55 | 0 | 57.2 | 19.1 |
| Hirundo_rustica3 | Brønderslev (Denmark) | 4.37 | 0.48 | 4 | 1 | 57.2 | 19.1 |
| Hirundo_rustica4 | Rovaniemi (Finland) | 11.18 |  | 1 | 0 | 66.45 | 19.1 |
| Hirundo_rustica5 | Granada (Spain) | 5 |  | 1 | 1 | 37.25 | 19.1 |
| Hirundo_rustica6 | Budapest (Hungary) | 5.5 | 0.71 | 2 | 1 | 47.45 | 19.1 |
| Hirundo_rustica7 | Poznan (Poland) | 10.77 | 5.26 | 8 | 0 | 52.45 | 19.1 |
| Hirundo_rustica8 | Toledo (Spain) | 12.78 | 8.9 | 9 | 1 | 39.83 | 19.1 |
| Lagopus_mutus | Oslo (Norway) | 9.57 | 2.44 | 7 | 0 | 59.92 | 426.5 |
| Lanius_collurio1 | Olomouc (Czech Republic) | 21.31 | 12.34 | 18 | 0 | 49.55 | 30.7 |
| Lanius_collurio2 | Brønderslev (Denmark) | 17.39 | 10.45 | 6 | 0 | 57.2 | 30.7 |
| Lanius_collurio3 | Budapest (Hungary) | 11.91 | 1.04 | 2 | 0 | 47.45 | 30.7 |
| Lanius_collurio4 | Poznan (Poland) | 35.01 |  | 1 | 0 | 52.45 | 30.7 |
| Lanius_excubitor1 | Budapest (Hungary) | 13.65 | 2.1 | 2 | 0 | 47.45 | 66.9 |
| Lanius_excubitor2 | Poznan (Poland) | 22.36 |  | 1 | 0 | 52.45 | 66.9 |
| Lanius_excubitor3 | Toledo (Spain) | 31.05 |  | 1 | 0 | 39.83 | 66.9 |
| Lanius_senator | Granada (Spain) | 36.12 | 13.42 | 4 | 0 | 37.25 | 36 |
| Larus_argentatus1 | Brønderslev (Denmark) | 50.09 | 23.39 | 35 | 0 | 57.2 | 895 |
| Larus_argentatus2 | Brønderslev (Denmark) | 26 | 1.41 | 4 | 1 | 57.2 | 895 |
| Larus_argentatus3 | Paris (France) | 15.1 | 5.04 | 10 | 0 | 48.1 | 895 |
| Larus_argentatus4 | Oslo (Norway) | 17.62 | 4.35 | 5 | 0 | 59.92 | 895 |
| Larus_argentatus5 | Oslo (Norway) | 12.8 | 6.1 | 5 | 1 | 59.92 | 895 |
| Larus_canus1 | Brønderslev (Denmark) | 59.94 | 7.74 | 48 | 0 | 57.2 | 386.5 |
| Larus_canus2 | Oslo (Norway) | 14 | 6.56 | 3 | 0 | 59.92 | 386.5 |
| Larus_canus3 | Oslo (Norway) | 11 | 0.58 | 3 | 1 | 59.92 | 386.5 |
| Larus_graellsii1 | Brønderslev (Denmark) | 27.89 | 5.34 | 168 | 0 | 57.2 | 817.5 |
| Larus_graellsii2 | Paris (France) | 22 |  | 1 | 0 | 48.1 | 817.5 |
| Larus_graellsii3 | Oslo (Norway) | 7.5 | 0.71 | 2 | 1 | 59.92 | 817.5 |
| Larus_marinus1 | Brønderslev (Denmark) | 57.52 | 6.04 | 42 | 0 | 57.2 | 1599.5 |
| Larus_marinus2 | Brønderslev (Denmark) | 24.75 | 9.5 | 12 | 1 | 57.2 | 1599.5 |
| Larus_marinus3 | Oslo (Norway) | 19.26 | 18.7 | 3 | 1 | 59.92 | 1599.5 |
| Larus_ridibundus1 | Olomouc (Czech Republic) | 3.16 |  | 1 | 1 | 49.55 | 280.5 |
| Larus_ridibundus2 | Brønderslev (Denmark) | 37.75 | 12.59 | 8 | 0 | 57.2 | 280.5 |
| Larus_ridibundus3 | Rovaniemi (Finland) | 19.46 | 10.08 | 39 | 1 | 66.45 | 280.5 |
| Larus_ridibundus4 | Oslo (Norway) | 9.58 | 5.24 | 9 | 0 | 59.92 | 280.5 |
| Larus_ridibundus5 | Oslo (Norway) | 8.47 | 4.25 | 36 | 1 | 59.92 | 280.5 |
| Locustella_naevia1 | Olomouc (Czech Republic) | 7.22 | 2.6 | 2 | 0 | 49.55 | 12.7 |
| Locustella_naevia2 | Brønderslev (Denmark) | 15.03 | 4.23 | 2 | 0 | 57.2 | 12.7 |
| Loxia_curvirostra | Brønderslev (Denmark) | 4.74 | 0.37 | 2 | 0 | 57.2 | 40.6 |
| Lullula_arborea | Brønderslev (Denmark) | 8 | 3 | 3 | 0 | 57.2 | 30.1 |
| Luscinia_luscinia | Brønderslev (Denmark) | 15.89 | 5.61 | 4 | 0 | 57.2 | 25 |
| Luscinia_megarhynchos1 | Olomouc (Czech Republic) | 15.52 |  | 1 | 0 | 49.55 | 20.2 |
| Luscinia_megarhynchos2 | Granada (Spain) | 21.07 | 6.18 | 4 | 0 | 37.25 | 20.2 |
| Luscinia_megarhynchos3 | Budapest (Hungary) | 10.53 | 4.18 | 15 | 0 | 47.45 | 20.2 |
| Luscinia_megarhynchos4 | Budapest (Hungary) | 16.49 |  | 1 | 1 | 47.45 | 20.2 |
| Luscinia_megarhynchos5 | Poznan (Poland) | 46.01 |  | 1 | 0 | 52.45 | 20.2 |
| Luscinia_megarhynchos6 | Toledo (Spain) | 17.51 | 10.62 | 2 | 0 | 39.83 | 20.2 |
| Luscinia_svecica | Oslo (Norway) | 11.33 | 4.16 | 3 | 0 | 59.92 | 18.3 |
| Mergus_merganser | Oslo (Norway) | 37.5 | 10.61 | 2 | 0 | 59.92 | 1641.5 |
| Mergus_serrator | Oslo (Norway) | 15 |  | 2 | 0 | 59.92 | 1090.5 |
| Merops_apiaster | Granada (Spain) | 94.95 | 38.68 | 13 | 0 | 37.25 | 55.1 |
| Miliaria_calandra1 | Brønderslev (Denmark) | 10.29 | 5.23 | 28 | 0 | 57.2 | 47.7 |
| Miliaria_calandra2 | Granada (Spain) | 25.2 | 14.26 | 12 | 0 | 37.25 | 47.7 |
| Miliaria_calandra3 | Poznan (Poland) | 31.64 | 15.28 | 7 | 0 | 52.45 | 47.7 |
| Miliaria_calandra4 | Toledo (Spain) | 15.69 | 8.77 | 14 | 0 | 39.83 | 47.7 |
| Miliaria_calandra5 | Toledo (Spain) | 10.52 | 6.39 | 2 | 1 | 39.83 | 47.7 |
| Motacilla_alba1 | Olomouc (Czech Republic) | 18.03 | 6.88 | 25 | 0 | 49.55 | 20.8 |
| Motacilla_alba10 | Granada (Spain) | 18.5 | 11.56 | 4 | 1 | 37.25 | 20.8 |
| Motacilla_alba11 | Oslo (Norway) | 7.15 | 3.04 | 47 | 0 | 59.92 | 20.8 |
| Motacilla_alba12 | Oslo (Norway) | 5.49 | 2.26 | 49 | 1 | 59.92 | 20.8 |
| Motacilla_alba13 | Poznan (Poland) | 25.56 | 17.13 | 8 | 0 | 52.45 | 20.8 |
| Motacilla_alba14 | Poznan (Poland) | 2 |  | 1 | 1 | 52.45 | 20.8 |
| Motacilla_alba15 | Toledo (Spain) | 8 |  | 1 | 0 | 39.83 | 20.8 |
| Motacilla_alba2 | Olomouc (Czech Republic) | 10 |  | 1 | 1 | 49.55 | 20.8 |
| Motacilla_alba3 | Brønderslev (Denmark) | 11.62 | 7.08 | 66 | 0 | 57.2 | 20.8 |
| Motacilla_alba4 | Brønderslev (Denmark) | 8.8 | 3.03 | 5 | 1 | 57.2 | 20.8 |
| Motacilla_alba5 | Rovaniemi (Finland) | 7.57 | 3.51 | 7 | 0 | 66.45 | 20.8 |
| Motacilla_alba6 | Rovaniemi (Finland) | 8.25 | 4.3 | 127 | 1 | 66.45 | 20.8 |
| Motacilla_alba7 | Paris (France) | 6.33 | 1.53 | 3 | 0 | 48.1 | 20.8 |
| Motacilla_alba8 | Paris (France) | 7 | 1.73 | 3 | 1 | 48.1 | 20.8 |
| Motacilla_alba9 | Granada (Spain) | 40.36 | 21.68 | 14 | 0 | 37.25 | 20.8 |
| Motacilla_cinerea1 | Olomouc (Czech Republic) | 8 |  | 1 | 0 | 49.55 | 17.4 |
| Motacilla_cinerea2 | Olomouc (Czech Republic) | 8.35 | 2.33 | 2 | 1 | 49.55 | 17.4 |
| Motacilla_cinerea3 | Brønderslev (Denmark) | 6.87 | 1.34 | 7 | 0 | 57.2 | 17.4 |
| Motacilla_cinerea4 | Paris (France) | 9.07 | 3.19 | 7 | 0 | 48.1 | 17.4 |
| Motacilla_cinerea5 | Paris (France) | 7.79 | 3.61 | 20 | 1 | 48.1 | 17.4 |
| Motacilla_flava1 | Brønderslev (Denmark) | 11.2 | 4.37 | 19 | 0 | 57.2 | 17.5 |
| Motacilla_flava2 | Rovaniemi (Finland) | 11.84 | 6.8 | 3 | 0 | 66.45 | 17.5 |
| Motacilla_flava3 | Rovaniemi (Finland) | 17 |  | 1 | 1 | 66.45 | 17.5 |
| Motacilla_flava4 | Oslo (Norway) | 18 |  | 1 | 0 | 59.92 | 17.5 |
| Motacilla_flava5 | Poznan (Poland) | 5.39 |  | 1 | 0 | 52.45 | 17.5 |
| Muscicapa_striata1 | Olomouc (Czech Republic) | 15.56 | 7.21 | 4 | 0 | 49.55 | 15.5 |
| Muscicapa_striata2 | Olomouc (Czech Republic) | 6.91 | 3.9 | 21 | 1 | 49.55 | 15.5 |
| Muscicapa_striata3 | Brønderslev (Denmark) | 10.08 | 2.52 | 12 | 0 | 57.2 | 15.5 |
| Muscicapa_striata4 | Brønderslev (Denmark) | 6.32 |  | 1 | 1 | 57.2 | 15.5 |
| Muscicapa_striata5 | Rovaniemi (Finland) | 9.06 |  | 1 | 0 | 66.45 | 15.5 |
| Muscicapa_striata6 | Paris (France) | 5.66 |  | 1 | 1 | 48.1 | 15.5 |
| Muscicapa_striata7 | Granada (Spain) | 27.45 |  | 1 | 0 | 37.25 | 15.5 |
| Muscicapa_striata8 | Granada (Spain) | 13.25 | 7.98 | 18 | 1 | 37.25 | 15.5 |
| Muscicapa_striata9 | Toledo (Spain) | 30.07 |  | 1 | 0 | 39.83 | 15.5 |
| Nucifraga_caryocatactes | Oslo (Norway) | 8.41 | 0.89 | 3 | 0 | 59.92 | 193 |
| Numenius_arquata1 | Brønderslev (Denmark) | 59.33 | 12.29 | 6 | 0 | 57.2 | 725 |
| Numenius_arquata2 | Rovaniemi (Finland) | 42.07 | 23.78 | 5 | 0 | 66.45 | 725 |
| Oenanthe_oenanthe1 | Brønderslev (Denmark) | 16.67 | 6.38 | 18 | 0 | 57.2 | 24 |
| Oenanthe_oenanthe2 | Oslo (Norway) | 19.78 | 3.35 | 9 | 0 | 59.92 | 24 |
| Oriolus_oriolus1 | Granada (Spain) | 38.59 | 32.62 | 2 | 0 | 37.25 | 68.5 |
| Oriolus_oriolus2 | Budapest (Hungary) | 24.12 | 11.15 | 5 | 0 | 47.45 | 68.5 |
| Oriolus_oriolus3 | Toledo (Spain) | 28.31 | 4.68 | 2 | 0 | 39.83 | 68.5 |
| Parus_ater1 | Olomouc (Czech Republic) | 12.08 |  | 1 | 0 | 49.55 | 9.3 |
| Parus_ater2 | Brønderslev (Denmark) | 5.41 | 1.93 | 8 | 0 | 57.2 | 9.3 |
| Parus_ater3 | Paris (France) | 4 |  | 1 | 0 | 48.1 | 9.3 |
| Parus_ater4 | Paris (France) | 5.66 |  | 1 | 1 | 48.1 | 9.3 |
| Parus_ater5 | Granada (Spain) | 18.49 | 9.32 | 3 | 0 | 37.25 | 9.3 |
| Parus_ater6 | Granada (Spain) | 5.77 | 3.56 | 7 | 1 | 37.25 | 9.3 |
| Parus_ater7 | Budapest (Hungary) | 7.69 | 3.26 | 2 | 0 | 47.45 | 9.3 |
| Parus_ater8 | Budapest (Hungary) | 3.92 | 0.45 | 2 | 1 | 47.45 | 9.3 |
| Parus_ater9 | Oslo (Norway) | 6 |  | 1 | 1 | 59.92 | 9.3 |
| Parus_caeruleus1 | Olomouc (Czech Republic) | 10.08 | 5.06 | 14 | 0 | 49.55 | 11.8 |
| Parus_caeruleus10 | Granada (Spain) | 7.3 | 5.23 | 2 | 1 | 37.25 | 11.8 |
| Parus_caeruleus11 | Budapest (Hungary) | 5.94 | 0.92 | 11 | 0 | 47.45 | 11.8 |
| Parus_caeruleus12 | Budapest (Hungary) | 7.05 | 2.42 | 11 | 1 | 47.45 | 11.8 |
| Parus_caeruleus13 | Oslo (Norway) | 6.09 | 3.72 | 16 | 0 | 59.92 | 11.8 |
| Parus_caeruleus14 | Oslo (Norway) | 4.18 | 1.53 | 14 | 1 | 59.92 | 11.8 |
| Parus_caeruleus15 | Toledo (Spain) | 16.44 | 6.88 | 11 | 0 | 39.83 | 11.8 |
| Parus_caeruleus16 | Toledo (Spain) | 10.55 | 1.54 | 5 | 1 | 39.83 | 11.8 |
| Parus_caeruleus2 | Olomouc (Czech Republic) | 4.14 | 1.18 | 7 | 1 | 49.55 | 11.8 |
| Parus_caeruleus3 | Brønderslev (Denmark) | 6.61 | 1.74 | 23 | 0 | 57.2 | 11.8 |
| Parus_caeruleus4 | Brønderslev (Denmark) | 3.72 | 0.8 | 44 | 1 | 57.2 | 11.8 |
| Parus_caeruleus5 | Rovaniemi (Finland) | 8 |  | 1 | 0 | 66.45 | 11.8 |
| Parus_caeruleus6 | Rovaniemi (Finland) | 3.12 | 1.25 | 2 | 1 | 66.45 | 11.8 |
| Parus_caeruleus7 | Paris (France) | 5.17 | 1.29 | 28 | 0 | 48.1 | 11.8 |
| Parus_caeruleus8 | Paris (France) | 4.78 | 1.69 | 43 | 1 | 48.1 | 11.8 |
| Parus_caeruleus9 | Granada (Spain) | 17.75 | 7.2 | 23 | 0 | 37.25 | 11.8 |
| Parus_cristatus1 | Brønderslev (Denmark) | 7.2 | 2.7 | 11 | 0 | 57.2 | 11.2 |
| Parus_cristatus2 | Paris (France) | 4.81 | 2.08 | 3 | 0 | 48.1 | 11.2 |
| Parus_cristatus3 | Paris (France) | 6.04 | 1.45 | 6 | 1 | 48.1 | 11.2 |
| Parus_major1 | Olomouc (Czech Republic) | 10.47 | 3.97 | 25 | 0 | 49.55 | 18.5 |
| Parus_major10 | Budapest (Hungary) | 7.65 | 7.48 | 68 | 0 | 47.45 | 18.5 |
| Parus_major11 | Budapest (Hungary) | 5.4 | 1.83 | 86 | 1 | 47.45 | 18.5 |
| Parus_major12 | Oslo (Norway) | 5.74 | 2.64 | 30 | 0 | 59.92 | 18.5 |
| Parus_major13 | Oslo (Norway) | 4.4 | 1.55 | 26 | 1 | 59.92 | 18.5 |
| Parus_major14 | Poznan (Poland) | 16.49 |  | 1 | 0 | 52.45 | 18.5 |
| Parus_major15 | Poznan (Poland) | 3.66 | 0.98 | 7 | 1 | 52.45 | 18.5 |
| Parus_major16 | Toledo (Spain) | 11.39 | 5.87 | 13 | 0 | 39.83 | 18.5 |
| Parus_major17 | Toledo (Spain) | 8.39 | 3.83 | 16 | 1 | 39.83 | 18.5 |
| Parus_major2 | Olomouc (Czech Republic) | 3.62 | 1.7 | 15 | 1 | 49.55 | 18.5 |
| Parus_major3 | Brønderslev (Denmark) | 5.46 | 2.52 | 51 | 0 | 57.2 | 18.5 |
| Parus_major4 | Brønderslev (Denmark) | 4.89 | 1.86 | 29 | 1 | 57.2 | 18.5 |
| Parus_major5 | Rovaniemi (Finland) | 8.61 | 0.86 | 2 | 0 | 66.45 | 18.5 |
| Parus_major6 | Rovaniemi (Finland) | 5.24 | 3.35 | 12 | 1 | 66.45 | 18.5 |
| Parus_major7 | Paris (France) | 4.6 | 1.59 | 42 | 0 | 48.1 | 18.5 |
| Parus_major8 | Paris (France) | 4.75 | 1.24 | 68 | 1 | 48.1 | 18.5 |
| Parus_major9 | Granada (Spain) | 22.38 | 13.44 | 28 | 0 | 37.25 | 18.5 |
| Parus_montanus | Oslo (Norway) | 5.61 | 2.82 | 7 | 0 | 59.92 | 11.7 |
| Parus_palustris1 | Olomouc (Czech Republic) | 10.6 | 2.9 | 2 | 0 | 49.55 | 11.9 |
| Parus_palustris2 | Brønderslev (Denmark) | 4.03 | 1.33 | 12 | 0 | 57.2 | 11.9 |
| Parus_palustris3 | Paris (France) | 6.6 | 1.96 | 4 | 0 | 48.1 | 11.9 |
| Parus_palustris4 | Paris (France) | 6.1 | 3.42 | 6 | 1 | 48.1 | 11.9 |
| Passer_domesticus1 | Olomouc (Czech Republic) | 15.28 | 6.34 | 20 | 0 | 49.55 | 30.4 |
| Passer_domesticus10 | Granada (Spain) | 10.33 | 9.49 | 328 | 1 | 37.25 | 30.4 |
| Passer_domesticus11 | Budapest (Hungary) | 7.34 | 2.83 | 54 | 0 | 47.45 | 30.4 |
| Passer_domesticus12 | Budapest (Hungary) | 7.36 | 3.34 | 17 | 1 | 47.45 | 30.4 |
| Passer_domesticus13 | Oslo (Norway) | 4.24 |  | 2 | 0 | 59.92 | 30.4 |
| Passer_domesticus14 | Oslo (Norway) | 3.06 | 1.17 | 71 | 1 | 59.92 | 30.4 |
| Passer_domesticus15 | Poznan (Poland) | 11.05 | 7.42 | 21 | 0 | 52.45 | 30.4 |
| Passer_domesticus16 | Poznan (Poland) | 3.49 | 2.48 | 26 | 1 | 52.45 | 30.4 |
| Passer_domesticus17 | Toledo (Spain) | 15.84 | 6.57 | 30 | 0 | 39.83 | 30.4 |
| Passer_domesticus18 | Toledo (Spain) | 11.85 | 6.8 | 78 | 1 | 39.83 | 30.4 |
| Passer_domesticus2 | Olomouc (Czech Republic) | 3.59 | 1.11 | 16 | 1 | 49.55 | 30.4 |
| Passer_domesticus3 | Brønderslev (Denmark) | 4.72 | 3.29 | 18 | 0 | 57.2 | 30.4 |
| Passer_domesticus4 | Brønderslev (Denmark) | 4.52 | 1.82 | 19 | 1 | 57.2 | 30.4 |
| Passer_domesticus5 | Rovaniemi (Finland) | 16.67 | 8.39 | 3 | 0 | 66.45 | 30.4 |
| Passer_domesticus6 | Rovaniemi (Finland) | 4.37 | 4.05 | 34 | 1 | 66.45 | 30.4 |
| Passer_domesticus7 | Paris (France) | 4.83 | 2.05 | 11 | 0 | 48.1 | 30.4 |
| Passer_domesticus8 | Paris (France) | 3.38 | 1.53 | 122 | 1 | 48.1 | 30.4 |
| Passer_domesticus9 | Granada (Spain) | 47.69 | 21.62 | 193 | 0 | 37.25 | 30.4 |
| Passer_hispaniolensis | Toledo (Spain) | 20.21 | 0.26 | 2 | 0 | 39.83 | 26.6 |
| Passer_montanus1 | Olomouc (Czech Republic) | 16.67 | 7.38 | 18 | 0 | 49.55 | 21.7 |
| Passer_montanus10 | Oslo (Norway) | 4.62 | 1.38 | 67 | 1 | 59.92 | 21.7 |
| Passer_montanus11 | Poznan (Poland) | 14.45 | 2.99 | 5 | 0 | 52.45 | 21.7 |
| Passer_montanus12 | Poznan (Poland) | 5.52 | 3.48 | 11 | 1 | 52.45 | 21.7 |
| Passer_montanus13 | Toledo (Spain) | 12.32 | 4.07 | 13 | 0 | 39.83 | 21.7 |
| Passer_montanus14 | Toledo (Spain) | 13.19 | 8.03 | 20 | 1 | 39.83 | 21.7 |
| Passer_montanus2 | Olomouc (Czech Republic) | 2.78 | 1.14 | 11 | 1 | 49.55 | 21.7 |
| Passer_montanus3 | Brønderslev (Denmark) | 6.35 | 2.6 | 24 | 0 | 57.2 | 21.7 |
| Passer_montanus4 | Brønderslev (Denmark) | 4.77 | 2.88 | 36 | 1 | 57.2 | 21.7 |
| Passer_montanus5 | Rovaniemi (Finland) | 4.23 | 3.26 | 5 | 1 | 66.45 | 21.7 |
| Passer_montanus6 | Granada (Spain) | 27.11 | 11.93 | 36 | 0 | 37.25 | 21.7 |
| Passer_montanus7 | Budapest (Hungary) | 8.08 | 3.12 | 45 | 0 | 47.45 | 21.7 |
| Passer_montanus8 | Budapest (Hungary) | 6.18 | 1.95 | 26 | 1 | 47.45 | 21.7 |
| Passer_montanus9 | Oslo (Norway) | 6.18 | 1.29 | 9 | 0 | 59.92 | 21.7 |
| Perdix_perdix | Brønderslev (Denmark) | 24.92 | 17.34 | 24 | 0 | 57.2 | 382 |
| Phalacrocorax_carbo1 | Brønderslev (Denmark) | 68.67 | 33.4 | 12 | 0 | 57.2 | 2254 |
| Phalacrocorax_carbo2 | Oslo (Norway) | 39.5 | 0.71 | 2 | 0 | 59.92 | 2254 |
| Phalacrocorax_carbo3 | Oslo (Norway) | 7 | 1.41 | 2 | 1 | 59.92 | 2254 |
| Phasianus_colchicus1 | Olomouc (Czech Republic) | 30 |  | 2 | 0 | 49.55 | 1400 |
| Phasianus_colchicus2 | Olomouc (Czech Republic) | 12 |  | 1 | 1 | 49.55 | 1400 |
| Phasianus_colchicus3 | Brønderslev (Denmark) | 18.5 | 16.53 | 6 | 0 | 57.2 | 1400 |
| Phasianus_colchicus4 | Budapest (Hungary) | 26.33 | 9.3 | 12 | 0 | 47.45 | 1400 |
| Phasianus_colchicus5 | Poznan (Poland) | 17.2 | 11.29 | 2 | 0 | 52.45 | 1400 |
| Phoenicurus_ochruros1 | Olomouc (Czech Republic) | 15.89 | 8.07 | 21 | 0 | 49.55 | 16 |
| Phoenicurus_ochruros10 | Toledo (Spain) | 16.16 |  | 1 | 1 | 39.83 | 16 |
| Phoenicurus_ochruros2 | Olomouc (Czech Republic) | 6.1 | 4.76 | 43 | 1 | 49.55 | 16 |
| Phoenicurus_ochruros3 | Brønderslev (Denmark) | 7.21 |  | 1 | 0 | 57.2 | 16 |
| Phoenicurus_ochruros4 | Paris (France) | 4.12 |  | 1 | 0 | 48.1 | 16 |
| Phoenicurus_ochruros5 | Paris (France) | 6.29 | 3.47 | 6 | 1 | 48.1 | 16 |
| Phoenicurus_ochruros6 | Budapest (Hungary) | 15.1 | 7.88 | 27 | 0 | 47.45 | 16 |
| Phoenicurus_ochruros7 | Budapest (Hungary) | 12.9 | 5.05 | 7 | 1 | 47.45 | 16 |
| Phoenicurus_ochruros8 | Poznan (Poland) | 22.4 | 12.86 | 8 | 0 | 52.45 | 16 |
| Phoenicurus_ochruros9 | Poznan (Poland) | 3.16 |  | 1 | 1 | 52.45 | 16 |
| Phoenicurus_phoenicurus1 | Olomouc (Czech Republic) | 19.1 |  | 1 | 0 | 49.55 | 15.9 |
| Phoenicurus_phoenicurus2 | Olomouc (Czech Republic) | 5.11 | 1.38 | 3 | 1 | 49.55 | 15.9 |
| Phoenicurus_phoenicurus3 | Brønderslev (Denmark) | 12.05 | 7.12 | 9 | 0 | 57.2 | 15.9 |
| Phoenicurus_phoenicurus4 | Brønderslev (Denmark) | 9.23 | 2.39 | 11 | 1 | 57.2 | 15.9 |
| Phoenicurus_phoenicurus5 | Rovaniemi (Finland) | 20.02 |  | 1 | 0 | 66.45 | 15.9 |
| Phoenicurus_phoenicurus6 | Rovaniemi (Finland) | 3 |  | 1 | 1 | 66.45 | 15.9 |
| Phoenicurus_phoenicurus7 | Budapest (Hungary) | 21.1 |  | 1 | 0 | 47.45 | 15.9 |
| Phylloscopus_collybita1 | Olomouc (Czech Republic) | 11.03 | 3.87 | 7 | 0 | 49.55 | 7.7 |
| Phylloscopus_collybita2 | Olomouc (Czech Republic) | 4.31 | 1.25 | 4 | 1 | 49.55 | 7.7 |
| Phylloscopus_collybita3 | Brønderslev (Denmark) | 7.88 | 2.22 | 15 | 0 | 57.2 | 7.7 |
| Phylloscopus_collybita4 | Brønderslev (Denmark) | 5.05 | 0.8 | 5 | 1 | 57.2 | 7.7 |
| Phylloscopus_collybita5 | Paris (France) | 5.25 | 1.09 | 8 | 0 | 48.1 | 7.7 |
| Phylloscopus_collybita6 | Paris (France) | 5.49 | 1.77 | 7 | 1 | 48.1 | 7.7 |
| Phylloscopus_collybita7 | Granada (Spain) | 3.61 |  | 1 | 1 | 37.25 | 7.7 |
| Phylloscopus_collybita8 | Budapest (Hungary) | 4.12 |  | 1 | 1 | 47.45 | 7.7 |
| Phylloscopus_collybita9 | Toledo (Spain) | 10.59 | 0.83 | 2 | 1 | 39.83 | 7.7 |
| Phylloscopus_trochilus1 | Brønderslev (Denmark) | 6.06 | 1.99 | 82 | 0 | 57.2 | 9.4 |
| Phylloscopus_trochilus2 | Brønderslev (Denmark) | 6.11 | 1.57 | 6 | 1 | 57.2 | 9.4 |
| Phylloscopus_trochilus3 | Budapest (Hungary) | 2 |  | 1 | 0 | 47.45 | 9.4 |
| Phylloscopus_trochilus4 | Oslo (Norway) | 5.05 | 0.07 | 2 | 0 | 59.92 | 9.4 |
| Pica_pica1 | Olomouc (Czech Republic) | 20 | 7.43 | 6 | 1 | 49.55 | 228 |
| Pica_pica10 | Budapest (Hungary) | 21.46 | 6.48 | 37 | 0 | 47.45 | 228 |
| Pica_pica11 | Budapest (Hungary) | 10.26 | 6.89 | 2 | 1 | 47.45 | 228 |
| Pica_pica12 | Oslo (Norway) | 13.98 | 5.63 | 35 | 0 | 59.92 | 228 |
| Pica_pica13 | Oslo (Norway) | 8.3 | 4.48 | 60 | 1 | 59.92 | 228 |
| Pica_pica14 | Poznan (Poland) | 26.66 | 16.96 | 14 | 0 | 52.45 | 228 |
| Pica_pica15 | Poznan (Poland) | 6.55 | 4.49 | 44 | 1 | 52.45 | 228 |
| Pica_pica16 | Toledo (Spain) | 31.53 | 13.34 | 17 | 0 | 39.83 | 228 |
| Pica_pica17 | Toledo (Spain) | 16.64 | 7.52 | 14 | 1 | 39.83 | 228 |
| Pica_pica2 | Brønderslev (Denmark) | 37.59 | 26.44 | 16 | 0 | 57.2 | 228 |
| Pica_pica3 | Brønderslev (Denmark) | 10.39 | 5.92 | 16 | 1 | 57.2 | 228 |
| Pica_pica4 | Rovaniemi (Finland) | 29 |  | 1 | 0 | 66.45 | 228 |
| Pica_pica5 | Rovaniemi (Finland) | 15.36 | 9.29 | 31 | 1 | 66.45 | 228 |
| Pica_pica6 | Paris (France) | 15.12 | 7.81 | 66 | 0 | 48.1 | 228 |
| Pica_pica7 | Paris (France) | 8.79 | 4.75 | 88 | 1 | 48.1 | 228 |
| Pica_pica8 | Granada (Spain) | 78.95 | 43.83 | 85 | 0 | 37.25 | 228 |
| Pica_pica9 | Granada (Spain) | 25.94 | 12.5 | 19 | 1 | 37.25 | 228 |
| Picoides_syriacus | Budapest (Hungary) | 13.99 | 9.42 | 6 | 0 | 47.45 | 76.8 |
| Picus_canus | Olomouc (Czech Republic) | 19 |  | 1 | 1 | 49.55 | 136.5 |
| Picus_viridis1 | Paris (France) | 17.82 | 6.14 | 19 | 0 | 48.1 | 193.5 |
| Picus_viridis2 | Paris (France) | 15.95 | 7.45 | 11 | 1 | 48.1 | 193.5 |
| Picus_viridis3 | Budapest (Hungary) | 7.07 |  | 1 | 1 | 47.45 | 193.5 |
| Picus_viridis4 | Oslo (Norway) | 6.71 |  | 1 | 0 | 59.92 | 193.5 |
| Picus_viridis5 | Poznan (Poland) | 18.68 |  | 1 | 1 | 52.45 | 193.5 |
| Picus_viridis6 | Toledo (Spain) | 50.24 |  | 1 | 0 | 39.83 | 193.5 |
| Pluvialis_apricaria1 | Brønderslev (Denmark) | 47 |  | 1 | 0 | 57.2 | 175.5 |
| Pluvialis_apricaria2 | Rovaniemi (Finland) | 18 |  | 1 | 0 | 66.45 | 175.5 |
| Prunella_modularis1 | Olomouc (Czech Republic) | 13.56 | 3.51 | 6 | 0 | 49.55 | 19 |
| Prunella_modularis2 | Brønderslev (Denmark) | 7.43 | 5.66 | 9 | 0 | 57.2 | 19 |
| Prunella_modularis3 | Paris (France) | 4.61 | 1.68 | 20 | 0 | 48.1 | 19 |
| Prunella_modularis4 | Paris (France) | 4.11 | 1.3 | 30 | 1 | 48.1 | 19 |
| Pyrrhula_pyrrhula1 | Brønderslev (Denmark) | 7.65 | 1.41 | 6 | 0 | 57.2 | 31.1 |
| Pyrrhula_pyrrhula2 | Brønderslev (Denmark) | 4.61 | 0.69 | 2 | 1 | 57.2 | 31.1 |
| Pyrrhula_pyrrhula3 | Rovaniemi (Finland) | 5 | 1.73 | 3 | 0 | 66.45 | 31.1 |
| Pyrrhula_pyrrhula4 | Paris (France) | 4.83 | 1.49 | 6 | 1 | 48.1 | 31.1 |
| Pyrrhula_pyrrhula5 | Oslo (Norway) | 16 | 2.83 | 2 | 0 | 59.92 | 31.1 |
| Regulus_ignicapillus1 | Paris (France) | 4.56 | 0.88 | 9 | 1 | 48.1 | 5.3 |
| Regulus_ignicapillus2 | Granada (Spain) | 6.5 | 6.36 | 2 | 1 | 37.25 | 5.3 |
| Regulus_regulus1 | Brønderslev (Denmark) | 5.41 | 1.45 | 6 | 0 | 57.2 | 5.8 |
| Regulus_regulus2 | Paris (France) | 3.24 | 1.75 | 2 | 0 | 48.1 | 5.8 |
| Regulus_regulus3 | Paris (France) | 3.28 | 1.21 | 27 | 1 | 48.1 | 5.8 |
| Regulus_regulus4 | Oslo (Norway) | 5.11 | 1.17 | 3 | 0 | 59.92 | 5.8 |
| Regulus_regulus5 | Oslo (Norway) | 4 |  | 1 | 1 | 59.92 | 5.8 |
| Riparia_riparia | Brønderslev (Denmark) | 23.1 | 7.44 | 7 | 0 | 57.2 | 13.2 |
| Rissa_tridactyla1 | Brønderslev (Denmark) | 35 |  | 2 | 0 | 57.2 | 407 |
| Rissa_tridactyla2 | Brønderslev (Denmark) | 3 |  | 1 | 1 | 57.2 | 407 |
| Saxicola_rubetra1 | Brønderslev (Denmark) | 15.43 | 7.9 | 23 | 0 | 57.2 | 16.6 |
| Saxicola_rubetra2 | Rovaniemi (Finland) | 23.09 |  | 1 | 0 | 66.45 | 16.6 |
| Saxicola_torquata | Budapest (Hungary) | 15.98 | 6.58 | 4 | 0 | 47.45 | 14.9 |
| Scolopax_rusticola1 | Rovaniemi (Finland) | 9 |  | 1 | 0 | 66.45 | 309.5 |
| Scolopax_rusticola2 | Poznan (Poland) | 8.49 |  | 1 | 0 | 52.45 | 309.5 |
| Serinus_serinus1 | Olomouc (Czech Republic) | 13.94 | 5.46 | 7 | 0 | 49.55 | 12 |
| Serinus_serinus10 | Toledo (Spain) | 11.56 | 5.66 | 22 | 1 | 39.83 | 12 |
| Serinus_serinus2 | Olomouc (Czech Republic) | 6 | 2.25 | 15 | 1 | 49.55 | 12 |
| Serinus_serinus3 | Paris (France) | 6.06 | 1.64 | 17 | 0 | 48.1 | 12 |
| Serinus_serinus4 | Paris (France) | 4.06 | 1.15 | 18 | 1 | 48.1 | 12 |
| Serinus_serinus5 | Granada (Spain) | 26.84 | 11.73 | 151 | 0 | 37.25 | 12 |
| Serinus_serinus6 | Granada (Spain) | 7.32 | 4.41 | 64 | 1 | 37.25 | 12 |
| Serinus_serinus7 | Budapest (Hungary) | 14.14 |  | 1 | 0 | 47.45 | 12 |
| Serinus_serinus8 | Poznan (Poland) | 24.04 | 8.54 | 2 | 0 | 52.45 | 12 |
| Serinus_serinus9 | Toledo (Spain) | 15.54 | 6.51 | 18 | 0 | 39.83 | 12 |
| Sitta_europaea1 | Olomouc (Czech Republic) | 13.39 | 2.91 | 5 | 0 | 49.55 | 23.9 |
| Sitta_europaea2 | Olomouc (Czech Republic) | 3.22 | 0.81 | 11 | 1 | 49.55 | 23.9 |
| Sitta_europaea3 | Paris (France) | 4.92 | 1.5 | 5 | 0 | 48.1 | 23.9 |
| Sitta_europaea4 | Paris (France) | 9.21 | 7.25 | 11 | 1 | 48.1 | 23.9 |
| Sitta_europaea5 | Budapest (Hungary) | 6.63 | 4.34 | 4 | 1 | 47.45 | 23.9 |
| Sitta_europaea6 | Oslo (Norway) | 5.41 | 2.55 | 2 | 0 | 59.92 | 23.9 |
| Sitta_europaea7 | Oslo (Norway) | 4.33 | 1.07 | 6 | 1 | 59.92 | 23.9 |
| Streptopelia_decaocto1 | Olomouc (Czech Republic) | 22.42 | 11.3 | 9 | 0 | 49.55 | 201.5 |
| Streptopelia_decaocto10 | Poznan (Poland) | 13.31 | 8.69 | 4 | 0 | 52.45 | 201.5 |
| Streptopelia_decaocto11 | Poznan (Poland) | 18.68 |  | 1 | 1 | 52.45 | 201.5 |
| Streptopelia_decaocto12 | Toledo (Spain) | 27.71 | 6.55 | 6 | 0 | 39.83 | 201.5 |
| Streptopelia_decaocto13 | Toledo (Spain) | 13.16 | 5.66 | 10 | 1 | 39.83 | 201.5 |
| Streptopelia_decaocto2 | Olomouc (Czech Republic) | 7.33 | 2.96 | 7 | 1 | 49.55 | 201.5 |
| Streptopelia_decaocto3 | Brønderslev (Denmark) | 26.43 | 19.42 | 7 | 0 | 57.2 | 201.5 |
| Streptopelia_decaocto4 | Brønderslev (Denmark) | 7.7 | 2.63 | 16 | 1 | 57.2 | 201.5 |
| Streptopelia_decaocto5 | Paris (France) | 11.67 |  | 1 | 0 | 48.1 | 201.5 |
| Streptopelia_decaocto6 | Paris (France) | 3.66 | 1.79 | 38 | 1 | 48.1 | 201.5 |
| Streptopelia_decaocto7 | Granada (Spain) | 46.73 | 38.64 | 3 | 0 | 37.25 | 201.5 |
| Streptopelia_decaocto8 | Granada (Spain) | 10.69 | 7.95 | 36 | 1 | 37.25 | 201.5 |
| Streptopelia_decaocto9 | Budapest (Hungary) | 10.79 | 5.59 | 39 | 0 | 47.45 | 201.5 |
| Streptopelia_turtur1 | Olomouc (Czech Republic) | 27.99 | 17.62 | 2 | 0 | 49.55 | 136.5 |
| Streptopelia_turtur2 | Paris (France) | 7.07 |  | 1 | 0 | 48.1 | 136.5 |
| Streptopelia_turtur3 | Granada (Spain) | 97.53 | 69.96 | 18 | 0 | 37.25 | 136.5 |
| Streptopelia_turtur4 | Budapest (Hungary) | 5 |  | 1 | 1 | 47.45 | 136.5 |
| Sturnus_unicolor1 | Granada (Spain) | 65.3 | 37.3 | 42 | 0 | 37.25 | 90.6 |
| Sturnus_unicolor2 | Granada (Spain) | 20.61 | 13.01 | 22 | 1 | 37.25 | 90.6 |
| Sturnus_unicolor3 | Toledo (Spain) | 26.37 | 11 | 29 | 0 | 39.83 | 90.6 |
| Sturnus_unicolor4 | Toledo (Spain) | 20.38 | 6.04 | 24 | 1 | 39.83 | 90.6 |
| Sturnus_vulgaris1 | Olomouc (Czech Republic) | 36.44 | 20.42 | 12 | 0 | 49.55 | 80.5 |
| Sturnus_vulgaris10 | Oslo (Norway) | 5.8 | 1.92 | 5 | 1 | 59.92 | 80.5 |
| Sturnus_vulgaris11 | Poznan (Poland) | 31.28 | 20.74 | 10 | 0 | 52.45 | 80.5 |
| Sturnus_vulgaris12 | Poznan (Poland) | 5.2 | 3.6 | 15 | 1 | 52.45 | 80.5 |
| Sturnus_vulgaris2 | Olomouc (Czech Republic) | 8.67 | 1.15 | 3 | 1 | 49.55 | 80.5 |
| Sturnus_vulgaris3 | Brønderslev (Denmark) | 14.31 | 6.16 | 42 | 0 | 57.2 | 80.5 |
| Sturnus_vulgaris4 | Brønderslev (Denmark) | 11.43 | 4.99 | 10 | 1 | 57.2 | 80.5 |
| Sturnus_vulgaris5 | Paris (France) | 9.75 | 4.84 | 42 | 0 | 48.1 | 80.5 |
| Sturnus_vulgaris6 | Paris (France) | 6.4 | 2.92 | 56 | 1 | 48.1 | 80.5 |
| Sturnus_vulgaris7 | Budapest (Hungary) | 13.46 | 6.92 | 5 | 0 | 47.45 | 80.5 |
| Sturnus_vulgaris8 | Budapest (Hungary) | 14.5 | 6.16 | 12 | 1 | 47.45 | 80.5 |
| Sturnus_vulgaris9 | Oslo (Norway) | 8.83 | 7.66 | 6 | 0 | 59.92 | 80.5 |
| Sylvia_atricapilla1 | Olomouc (Czech Republic) | 13.56 | 6.49 | 13 | 0 | 49.55 | 18.9 |
| Sylvia_atricapilla10 | Budapest (Hungary) | 7.8 | 4.36 | 26 | 1 | 47.45 | 18.9 |
| Sylvia_atricapilla11 | Oslo (Norway) | 3.16 |  | 1 | 0 | 59.92 | 18.9 |
| Sylvia_atricapilla12 | Poznan (Poland) | 11.32 | 2.97 | 2 | 0 | 52.45 | 18.9 |
| Sylvia_atricapilla13 | Toledo (Spain) | 11.07 | 3.55 | 4 | 0 | 39.83 | 18.9 |
| Sylvia_atricapilla14 | Toledo (Spain) | 16.2 | 9.03 | 3 | 1 | 39.83 | 18.9 |
| Sylvia_atricapilla2 | Olomouc (Czech Republic) | 4.89 | 1.47 | 12 | 1 | 49.55 | 18.9 |
| Sylvia_atricapilla3 | Brønderslev (Denmark) | 8.48 | 3.77 | 11 | 0 | 57.2 | 18.9 |
| Sylvia_atricapilla4 | Brønderslev (Denmark) | 5.44 | 0.74 | 6 | 1 | 57.2 | 18.9 |
| Sylvia_atricapilla5 | Paris (France) | 4.83 | 1.55 | 10 | 0 | 48.1 | 18.9 |
| Sylvia_atricapilla6 | Paris (France) | 4.95 | 1.22 | 14 | 1 | 48.1 | 18.9 |
| Sylvia_atricapilla7 | Granada (Spain) | 20.43 | 9.75 | 8 | 0 | 37.25 | 18.9 |
| Sylvia_atricapilla8 | Granada (Spain) | 2.89 | 0.35 | 3 | 1 | 37.25 | 18.9 |
| Sylvia_atricapilla9 | Budapest (Hungary) | 10.81 | 5.65 | 11 | 0 | 47.45 | 18.9 |
| Sylvia_borin1 | Olomouc (Czech Republic) | 19.1 |  | 1 | 0 | 49.55 | 19.1 |
| Sylvia_borin2 | Brønderslev (Denmark) | 6.78 | 2.68 | 41 | 0 | 57.2 | 19.1 |
| Sylvia_borin3 | Brønderslev (Denmark) | 6.55 | 1.03 | 4 | 1 | 57.2 | 19.1 |
| Sylvia_cantillans1 | Toledo (Spain) | 5.22 | 1.56 | 2 | 0 | 39.83 | 8.1 |
| Sylvia_cantillans2 | Toledo (Spain) | 10.76 | 3.96 | 3 | 1 | 39.83 | 8.1 |
| Sylvia_communis1 | Olomouc (Czech Republic) | 14.7 | 7.34 | 12 | 0 | 49.55 | 14.5 |
| Sylvia_communis2 | Olomouc (Czech Republic) | 2.23 |  | 1 | 1 | 49.55 | 14.5 |
| Sylvia_communis3 | Brønderslev (Denmark) | 7.54 | 4.53 | 129 | 0 | 57.2 | 14.5 |
| Sylvia_communis4 | Brønderslev (Denmark) | 5.63 | 1 | 3 | 1 | 57.2 | 14.5 |
| Sylvia_communis5 | Granada (Spain) | 22.94 | 28.06 | 3 | 0 | 37.25 | 14.5 |
| Sylvia_communis6 | Poznan (Poland) | 10.2 |  | 1 | 0 | 52.45 | 14.5 |
| Sylvia_curruca1 | Olomouc (Czech Republic) | 17.26 |  | 1 | 0 | 49.55 | 12.4 |
| Sylvia_curruca2 | Olomouc (Czech Republic) | 2.83 | 2 | 2 | 1 | 49.55 | 12.4 |
| Sylvia_curruca3 | Brønderslev (Denmark) | 5.72 | 2.7 | 27 | 0 | 57.2 | 12.4 |
| Sylvia_curruca4 | Brønderslev (Denmark) | 4.64 | 0.58 | 6 | 1 | 57.2 | 12.4 |
| Sylvia_curruca5 | Budapest (Hungary) | 8.55 | 2.98 | 4 | 0 | 47.45 | 12.4 |
| Sylvia_curruca6 | Budapest (Hungary) | 5.29 | 2.64 | 3 | 1 | 47.45 | 12.4 |
| Sylvia_melanocephala1 | Granada (Spain) | 21.56 | 12.82 | 97 | 0 | 37.25 | 13.5 |
| Sylvia_melanocephala2 | Granada (Spain) | 8.05 | 6.08 | 8 | 1 | 37.25 | 13.5 |
| Sylvia_melanocephala3 | Toledo (Spain) | 10.39 | 8.42 | 3 | 0 | 39.83 | 13.5 |
| Sylvia_melanocephala4 | Toledo (Spain) | 6.4 |  | 1 | 1 | 39.83 | 13.5 |
| Tadorna_tadorna | Brønderslev (Denmark) | 37.71 | 13.45 | 14 | 0 | 57.2 | 1152 |
| Tringa_nebularia1 | Brønderslev (Denmark) | 37.75 | 13.57 | 4 | 0 | 57.2 | 173.5 |
| Tringa_nebularia2 | Oslo (Norway) | 30 |  | 1 | 0 | 59.92 | 173.5 |
| Tringa_ochropus | Brønderslev (Denmark) | 31 |  | 1 | 0 | 57.2 | 84.8 |
| Tringa_totanus | Brønderslev (Denmark) | 29.71 | 11.84 | 7 | 0 | 57.2 | 112 |
| Troglodytes_troglodytes1 | Olomouc (Czech Republic) | 10.55 | 0.7 | 2 | 0 | 49.55 | 8.9 |
| Troglodytes_troglodytes2 | Brønderslev (Denmark) | 7.51 | 3.93 | 17 | 0 | 57.2 | 8.9 |
| Troglodytes_troglodytes3 | Brønderslev (Denmark) | 5.34 | 1.14 | 11 | 1 | 57.2 | 8.9 |
| Troglodytes_troglodytes4 | Paris (France) | 4.9 | 1.66 | 16 | 0 | 48.1 | 8.9 |
| Troglodytes_troglodytes5 | Paris (France) | 4.11 | 1.49 | 25 | 1 | 48.1 | 8.9 |
| Troglodytes_troglodytes6 | Oslo (Norway) | 3 |  | 1 | 0 | 59.92 | 8.9 |
| Troglodytes_troglodytes7 | Oslo (Norway) | 4.1 | 2.6 | 3 | 1 | 59.92 | 8.9 |
| Turdus_iliacus1 | Olomouc (Czech Republic) | 28 |  | 1 | 1 | 49.55 | 62.9 |
| Turdus_iliacus2 | Brønderslev (Denmark) | 12.75 | 6.67 | 4 | 0 | 57.2 | 62.9 |
| Turdus_iliacus3 | Rovaniemi (Finland) | 14.05 | 10.68 | 8 | 0 | 66.45 | 62.9 |
| Turdus_iliacus4 | Rovaniemi (Finland) | 6.77 | 6.55 | 8 | 1 | 66.45 | 62.9 |
| Turdus_iliacus5 | Oslo (Norway) | 4 |  | 1 | 0 | 59.92 | 62.9 |
| Turdus_iliacus6 | Poznan (Poland) | 12.21 |  | 1 | 0 | 52.45 | 62.9 |
| Turdus_merula1 | Olomouc (Czech Republic) | 21.51 | 18.11 | 64 | 0 | 49.55 | 95.9 |
| Turdus_merula10 | Budapest (Hungary) | 6.63 | 3.34 | 144 | 1 | 47.45 | 95.9 |
| Turdus_merula11 | Oslo (Norway) | 9.65 | 4.77 | 19 | 0 | 59.92 | 95.9 |
| Turdus_merula12 | Oslo (Norway) | 6.31 | 2.67 | 40 | 1 | 59.92 | 95.9 |
| Turdus_merula13 | Poznan (Poland) | 22.59 | 17.36 | 47 | 0 | 52.45 | 95.9 |
| Turdus_merula14 | Poznan (Poland) | 5.31 | 6.02 | 61 | 1 | 52.45 | 95.9 |
| Turdus_merula15 | Toledo (Spain) | 19.89 | 8.84 | 25 | 0 | 39.83 | 95.9 |
| Turdus_merula16 | Toledo (Spain) | 10.53 | 5 | 48 | 1 | 39.83 | 95.9 |
| Turdus_merula2 | Olomouc (Czech Republic) | 4.02 | 3.41 | 337 | 1 | 49.55 | 95.9 |
| Turdus_merula3 | Brønderslev (Denmark) | 12.22 | 7.59 | 56 | 0 | 57.2 | 95.9 |
| Turdus_merula4 | Brønderslev (Denmark) | 5.79 | 3.19 | 83 | 1 | 57.2 | 95.9 |
| Turdus_merula5 | Paris (France) | 8.02 | 4.47 | 147 | 0 | 48.1 | 95.9 |
| Turdus_merula6 | Paris (France) | 5.72 | 3.15 | 163 | 1 | 48.1 | 95.9 |
| Turdus_merula7 | Granada (Spain) | 47.22 | 22.37 | 28 | 0 | 37.25 | 95.9 |
| Turdus_merula8 | Granada (Spain) | 9.56 | 6.66 | 173 | 1 | 37.25 | 95.9 |
| Turdus_merula9 | Budapest (Hungary) | 9.08 | 6.55 | 39 | 0 | 47.45 | 95.9 |
| Turdus_philomelos1 | Olomouc (Czech Republic) | 29.03 | 18.9 | 15 | 0 | 49.55 | 70.5 |
| Turdus_philomelos2 | Olomouc (Czech Republic) | 4.19 | 3.11 | 85 | 1 | 49.55 | 70.5 |
| Turdus_philomelos3 | Brønderslev (Denmark) | 15.37 | 11.83 | 11 | 0 | 57.2 | 70.5 |
| Turdus_philomelos4 | Paris (France) | 8.1 | 5.42 | 43 | 0 | 48.1 | 70.5 |
| Turdus_philomelos5 | Paris (France) | 4.56 | 1.77 | 31 | 1 | 48.1 | 70.5 |
| Turdus_philomelos6 | Poznan (Poland) | 19.03 |  | 1 | 1 | 52.45 | 70.5 |
| Turdus_pilaris1 | Olomouc (Czech Republic) | 29.31 | 20.07 | 15 | 0 | 49.55 | 92.1 |
| Turdus_pilaris10 | Poznan (Poland) | 5.5 | 4.47 | 27 | 1 | 52.45 | 92.1 |
| Turdus_pilaris2 | Olomouc (Czech Republic) | 10.29 | 4.72 | 7 | 1 | 49.55 | 92.1 |
| Turdus_pilaris3 | Brønderslev (Denmark) | 18.14 | 12.55 | 12 | 0 | 57.2 | 92.1 |
| Turdus_pilaris4 | Brønderslev (Denmark) | 6 | 1.41 | 2 | 1 | 57.2 | 92.1 |
| Turdus_pilaris5 | Rovaniemi (Finland) | 20.95 | 9.18 | 19 | 0 | 66.45 | 92.1 |
| Turdus_pilaris6 | Rovaniemi (Finland) | 12.42 | 6.43 | 53 | 1 | 66.45 | 92.1 |
| Turdus_pilaris7 | Oslo (Norway) | 8.56 | 3.12 | 19 | 0 | 59.92 | 92.1 |
| Turdus_pilaris8 | Oslo (Norway) | 8.64 | 4.63 | 91 | 1 | 59.92 | 92.1 |
| Turdus_pilaris9 | Poznan (Poland) | 5.5 | 0.71 | 2 | 0 | 52.45 | 92.1 |
| Turdus_viscivorus1 | Olomouc (Czech Republic) | 49.9 | 13.69 | 3 | 0 | 49.55 | 117.8 |
| Turdus_viscivorus2 | Brønderslev (Denmark) | 23.86 | 5.92 | 9 | 0 | 57.2 | 117.8 |
| Turdus_viscivorus3 | Brønderslev (Denmark) | 9 |  | 1 | 1 | 57.2 | 117.8 |
| Turdus_viscivorus4 | Paris (France) | 27 |  | 1 | 0 | 48.1 | 117.8 |
| Turdus_viscivorus5 | Paris (France) | 12.21 | 7.07 | 2 | 1 | 48.1 | 117.8 |
| Upupa_epops1 | Granada (Spain) | 56.21 | 25.37 | 27 | 0 | 37.25 | 67.1 |
| Upupa_epops2 | Toledo (Spain) | 35.01 | 30.43 | 3 | 0 | 39.83 | 67.1 |
| Upupa_epops3 | Toledo (Spain) | 20.25 | 7.42 | 2 | 1 | 39.83 | 67.1 |
| Uria_aalge | Oslo (Norway) | 30.07 |  | 1 | 0 | 59.92 | 861.5 |
| Vanellus_vanellus | Brønderslev (Denmark) | 33.99 | 16.84 | 72 | 0 | 57.2 | 218.5 |
