# Supplementary material for: The Geography of Fear: A Latitudinal Gradient in Anti-Predator Escape Distances of Birds across Europe
Source: PLoS One. 2013 May 28;8(5):e64634. doi: 10.1371/journal.pone.0064634 (PMC3665823; doi:10.1371/journal.pone.0064634)
Supplement: File S1 — R script for the phylogenetic generalized least square regression (PGLS) models. They were fitted to analyze relationships among FID, body mass, raptor abundance, latitude and habitat. (DOCX) [file pone.0064634.s002.docx]

**Supplementary file S1. R script for the phylogenetic generalized least square regression (PGLS) models.** They were fitted to analyze relationships among FID, body mass, raptor abundance, latitude and habitat.

library(MASS)

library(mvtnorm)

library(ape)

source("C:/pglm3.1.r")

setwd("C:/ ")

fa<-read.tree("Latitude_FID_Supplementary_File_2.txt")

V<-vcv.phylo(fa)

data<-read.table('Table_S1.txt',header=T)

attach(data)

fix(data)

mod1<-pglmEstLambda(LOG_FID ~ LOG_BODY_MASS + LOG_RAPTOR_ABUNDANCE + Latitude + Habitat + Latitude*Habitat, data = data , V)

summary(mod1)

mod2<-pglm(LOG_FID ~ LOG_BODY_MASS + Latitude + Habitat + Latitude*Habitat, data = data , V, lambda = **ESTIMATEDLAMBDA**)

summary(mod2)

anova(mod2)

(# **ESTIMATEDLAMBDA** is the value obtained in mod1 if stastistically different from 0)
